# US Cancer Mortality Trends Among Asian and Pacific Islander Populations

**DOI:** 10.1001/jamanetworkopen.2024.42451

**Published:** 2024-11-04

**Authors:** David T. Zhu, Isabella R. Pompa, David Qi, Saveli I. Goldberg, Richard J. Lee, Sophia C. Kamran

**Affiliations:** 1 Medical Scientist Training Program, School of Medicine, Virginia Commonwealth University, Richmond; 2 Department of Radiation Oncology, Massachusetts General Hospital, Boston; 3 Harvard Medical School, Boston, Massachusetts; 4 Department of Medical Oncology, Massachusetts General Hospital Cancer Center, Boston

## Abstract

**Importance:**

Cancer is the leading cause of death among Asian American individuals and the second leading cause of death among Native Hawaiian and Pacific Islander people.

**Objective:**

To evaluate longitudinal cancer mortality trends from 1999 to 2020 among Asian American and Pacific Islander populations in the US by demographic characteristics.

**Design, Setting, and Participants:**

This cross-sectional study used the Centers for Disease Control and Prevention Wide-Ranging Online Data for Epidemiologic Research database to obtain age-adjusted cancer death rates among Asian American and Pacific Islander individuals of all ages between January 1, 1999, and December 31, 2020. Data were analyzed from January 12 to March 19, 2024.

**Exposures:**

Age, sex, cancer type, and US census regions.

**Main Outcomes and Measures:**

Trends and average annual percent changes (AAPCs) in age-adjusted cancer-specific mortality (CSM) rates for non-Hispanic Asian American and Pacific Islander populations were estimated by cancer type, age, sex, and region using Joinpoint regression.

**Results:**

Between 1999 and 2020, 305 386 Asian American and Pacific Islander individuals (median [IQR] age, 69.5 [58.5-79.2] years; 51.1% male) died of cancer in the US. Overall, the CSM rate decreased by 1.5% annually. Men experienced a greater CSM rate decrease (AAPC, −1.8%; 95% CI, −2.2% to −1.3%) compared with women (AAPC, −1.1%; 95% CI: −1.2% to −1.0%). For women, death rates decreased for most cancer types but increased for uterine (AAPC, 2.5%; 95% CI, 2.0%-3.0%) and brain and central nervous system (AAPC, 1.4%; 95% CI: 0.7%-2.1%) cancers. Colorectal cancer mortality rates increased among men aged 45 to 54 years (AAPC, 1.3%; 95% CI, 0.5%-2.1%). Liver and intrahepatic bile duct cancer mortality increased for both men and women in all US census regions, uterine cancer mortality increased in all regions for women, and pancreatic cancer mortality increased in the Midwest for both men and women.

**Conclusions and Relevance:**

Although these findings show an overall decrease in CSM among Asian American and Pacific Islander populations, specific cancer types exhibited increased mortality rates, with further disparities by sex and age. Targeted, culturally adapted clinical and public health interventions are needed to narrow disparities in cancer mortality.

## Key Points

**Question:**

What were the trends in cancer mortality among Asian American and Pacific Islander populations from 1999 to 2020 in the US?

**Findings:**

In this cross-sectional study of mortality data from 305 386 Asian American and Pacific Islander individuals, uterine and brain and central nervous system cancer mortality rates increased in women from 1999 to 2020, and colorectal cancer mortality rates increased in men aged 45 to 54 years. In all US census regions, liver cancer mortality rates increased for both men and women.

**Meaning:**

These findings suggest that inequitable access to health care may contribute to disparities in cancer mortality trends within Asian American and Pacific Islander populations.

## Teaser

This cross-sectional study evaluates longitudinal cancer mortality trends among Asian American and Pacific Islander populations in the US by demographic characteristics.

## Introduction

Asian American and Pacific Islander individuals represent the fastest growing racial and ethnic demographic in the US and face a distinct cancer burden, with cancer surpassing heart disease as the leading cause of death for Asian American people and remaining the second leading cause of death among Native Hawaiian and Pacific Islander people.^1,2,3^ These trends are underscored by higher incidence rates of cancers with infectious etiologies, such as hepatitis B virus (HBV)–associated liver cancers, as well as with persistently low cancer screening rates and linguistic or health literacy barriers.^3^

Disparities in cancer outcomes among Asian American and Pacific Islander populations are fueled by a multifaceted interplay of social and structural determinants of health. Suboptimal cancer screening rates and limited access to both primary care and specialized oncology services contribute to prevalent inequities in cancer diagnosis and treatment.^3^ Additionally, variations in health literacy, English proficiency, socioeconomic status, immigration status, and dietary risk factors across specific Asian American and Pacific Islander ethnic groups further exacerbate these disparities in cancer incidence and mortality.^2,3^ These challenges highlight the need for more nuanced, culturally informed approaches to cancer screening, diagnosis, and treatment among Asian American and Pacific Islander populations.

In this study, we evaluated cancer-specific mortality (CSM) trends over 2 decades to comprehensively assess the burden of cancer among Asian American and Pacific Islander individuals. We aimed to broadly describe these trends both overall and by sex, age, and geographic variations across a wide range of cancer types, thereby informing more targeted clinical and public health interventions to address disparities in cancer mortality.

## Methods

In this cross-sectional study, we examined cancer mortality data from the Centers for Disease Control and Prevention Wide-Ranging Online Data for Epidemiologic Research (CDC WONDER) Underlying Cause of Death database between January 1, 1999, and December 31, 2020.^4,5^ The Mass General Brigham Institutional Review Board considered this study exempt as non–human participant research and waived the need for informed consent. The study followed the Strengthening the Reporting of Observational Studies in Epidemiology (STROBE) reporting guideline.

We used *International Statistical Classification of Diseases, Tenth Revision* codes to identify specific cancer types (eTable 1 in Supplement 1).^4,5^ Our study covered 18 specific cancer types, including bladder, brain and central nervous system (CNS), breast, cervical, colorectal, uterine, esophageal, kidney and renal pelvic, leukemia, liver and intrahepatic bile duct, lung and bronchial, myeloma, non-Hodgkin lymphoma, ovarian, pancreatic, prostate, stomach, and testicular.

The CDC WONDER database calculates age-adjusted mortality rate estimates using the direct method, applying age-specific mortality rates to the 2000 US standard population age distribution.^4^ Age-adjusted CSM rates were calculated per 100 000 population for non-Hispanic Asian American and Pacific Islander individuals both overall and by sociodemographic characteristics (ie, sex, cancer type, and US census region). We determined age-specific CSM rates for 4 US census regions (Northeast, Midwest, South, and West) and calculated crude age-specific CSM rates for each cancer type across the following age groups: 15 to 24, 25 to 34, 35 to 44, 45 to 54, 55 to 64, 65 to 74, 75 to 84, and 85 years or older.

### Statistical Analysis

The data analysis was performed between January 12 and March 19, 2024. Overall and cancer-specific CSM rates were analyzed using the National Cancer Institute’s Joinpoint Regression Program, version 4.9.1.0.7.^6,7^ Joinpoint regression is a type of piecewise linear regression that models time series data by fitting a series of contiguous linear segments, each with its own slope, thereby capturing changes in the trends of CSM rates over time.^6,7^ Joinpoint aims to determine the fewest statistically significant segments needed, and our analysis was constrained to a maximum of 4 join points, consistent with previously described methods.^5^

For each segment, the annual percent change (APC) was calculated as the slope of the linear regression line, expressed as a percentage, to reflect annual changes in CSM rates. The average APC (AAPC) from 1999 to 2020 was determined by taking a weighted average of these slopes, with weights corresponding to the length of the individual segments. We set statistical significance at a 2-sided *P* < .05 and used the Holm-Bonferroni correction to address the increased risk of type I errors due to multiple comparisons.^8^

## Results

**Figure 1.**
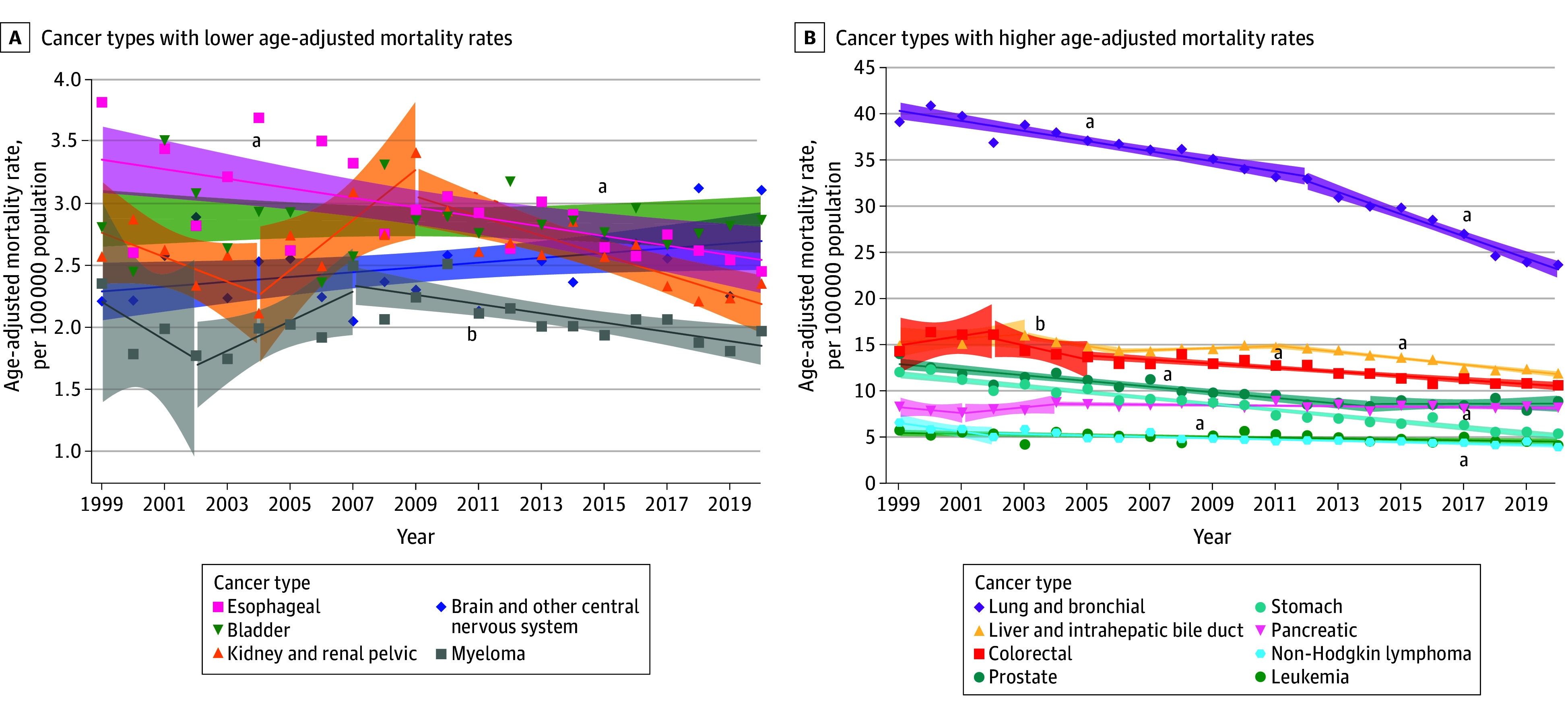
Trends in Age-Adjusted Mortality Rates Among Asian American and Pacific Islander Men by Cancer Type, 1999-2020 Markers indicate observed rates; shading, 95% CIs; solid lines, modeled trends. ^a^Statistically significant average annual percent change after Holm-Bonferroni correction. ^b^*P* < .05, but not significant by Holm-Bonferroni correction.

**Figure 2.**
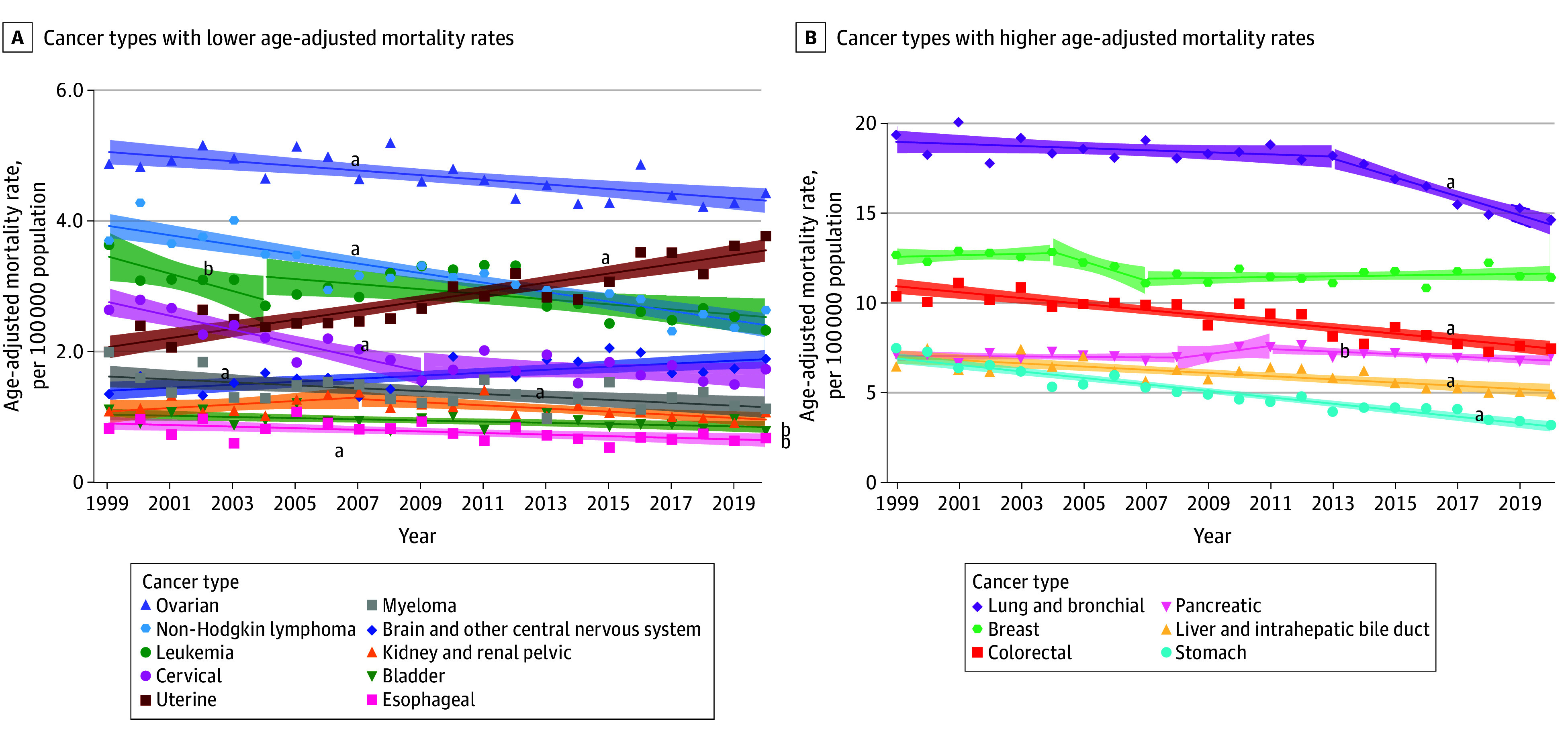
Trends in Age-Adjusted Mortality Rates Among Asian American and Pacific Islander Women by Cancer Type, 1999-2020 Markers indicate observed rates; shading, 95% CIs; solid lines, modeled trends. ^a^Statistically significant average annual percent change after Holm-Bonferroni correction. ^b^*P* < .05, but not significant by Holm-Bonferroni correction.

Between 1999 and 2020, 305 386 Asian American and Pacific Islander individuals (median [IQR], 69.5 [58.5-79.2] years; 48.9% female and 51.1% male) died of cancer in the US. Overall, CSM rates per 100 000 population were higher among men (123.8; 95% CI, 123.2-124.5) compared with women (89.3; 95% CI, 88.9-89.8) (eTable 2 in Supplement 1). The overall CSM rate decreased by 1.5% annually. Men experienced a greater reduction in cancer mortality during the study period (AAPC, −1.8%; 95% CI, −2.2% to −1.3%; *P* < .001) compared with women (AAPC, −1.1%; 95% CI, −1.2% to −1.0%; *P* < .001) (Table; eTable 3 in Supplement 1). For men, CSM rates decreased for all cancer sites, particularly lung and bronchial (AAPC, −2.6%; 95% CI, −2.9% to −2.3%; *P* < .001) and stomach (AAPC, −3.8%; 95% CI, −4.1% to −3.5%; *P* < .001) cancers, between 1999 and 2020 (Figure 1). While CSM rates from 1999 to 2020 declined for most cancer sites among women, significant increases were notably observed for uterine (AAPC, 2.5%; 95% CI, 2.0%-3.0%; *P* < .001) and brain and CNS cancers (AAPC, 1.4%; 95% CI, 0.7%-2.1%; *P* < .001) (Figure 2).

**Table.** AAPCs in Cancer Mortality Rates by Sex and Cancer Type Among Asian American and Pacific Islander Individuals, US 1999-2020^a^^,^^b^

Cause of death	All years		Segment 1			Segment 2			Segment 3			Segment 4		
	% (95% CI)	*P* value	Years	APC, % (95% CI)	*P* value	Years	APC, % (95% CI)	*P* value	Years	APC, % (95% CI)	*P* value	Years	APC, % (95% CI)	*P* value
All cancers	−1.5 (−1.7 to −1.2)	<.001	1999 to 2006	−1.6 (−1.9 to −1.2)	<.001	2006 to 2010	−0.5 (−1.9 to 0.9)	.47	2010 to 2020	−1.8 (−2.0 to −1.5)	<.001	NA	NA	NA
**Men**														
Overall cancer	−1.8 (−2.2 to −1.3)	<.001	1999 to 2006	−1.8 (−2.2 to −1.3)	<.001	2006 to 2009	−0.3 (−3.7 to 3.1)	.83	2009 to 2020	−2.1 (−2.3 to −1.9)	<.001	NA	NA	NA
Esophageal	−1.3 (−2.0 to −0.6)	.001	1999 to 2020	−1.3 (−2.0 to −0.6)	.001	NA	NA	NA	NA	NA	NA	NA	NA	NA
Stomach	−3.8 (−4.1 to −3.5)	<.001	1999 to 2020	−3.8 (−4.1 to −3.5)	<.001	NA	NA	NA	NA	NA	NA	NA	NA	NA
Colorectal	−1.6 (−3.1 to −0.1)	.04	1999 to 2001	6.4 (−2.4 to 16.1)	.14	2001 to 2005	−5.6 (−9.6 to −1.4)	.01	2005 to 2008	0.8 (−7.5 to 10.0)	.84	2008 to 2020	−2.2 (−2.7 to −1.7)	<.001
Liver and intrahepatic bile duct	−1.2 (−2.4 to 0.0)	.06	1999 to 2003	1.2 (−1.3 to 3.8)	.31	2003 to 2006	−3.9 (−11.3 to 4.1)	.29	2006 to 2011	1.0 (−1.5 to 3.6)	.41	2011 to 2020	−2.6 (−3.3 to −1.9)	<.001
Pancreatic	0.0 (−1.5 to 1.4)	.95	1999 to 2001	−4.1 (−12.4 to 5.0)	.34	2001 to 2004	4.1 (−4.9 to 14.0)	.35	2004 to 2020	−0.3 (−0.6 to 0.1)	.09	NA	NA	NA
Lung and bronchial	−2.6 (−2.9 to −2.3)	<.001	1999 to 2012	−1.5 (−1.9 to −1.1)	<.001	2012 to 2020	−4.3 (−5.1 to −3.6)	<.001	NA	NA	NA	NA	NA	NA
Myeloma	−0.8 (−2.9 to 1.4)	.50	1999 to 2002	−8.1 (−17.8 to 2.6)	.12	2002 to 2007	5.9 (−1.2 to 13.6)	.10	2007 to 2020	−1.5 (−2.6 to −0.3)	.02	NA	NA	NA
Kidney and renal pelvic	−1.0 (−2.9 to 0.9)	.30	1999 to 2004	−2.9 (−7.3 to 1.8)	.21	2004 to 2009	5.4 (−1.4 to 12.7)	.11	2009 to 2020	−3.0 (−4.3 to −1.6)	<.001	NA	NA	NA
Bladder	0.0 (−0.7 to 0.6)	.90	1999 to 2020	0.0 (−0.7 to 0.6)	.90	NA	NA	NA	NA	NA	NA	NA	NA	NA
Prostate	−1.9 (−2.8 to −1.1)	<.001	1999 to 2014	−2.8 (−3.5 to −2.1)	<.001	2014 to 2020	0.2 (−2.6 to 3.0)	.89	NA	NA	NA	NA	NA	NA
Brain and CNS	0.7 (0.0 to 1.5)	.050	1999 to 2020	0.7 (0.0 to 1.5)	.050	NA	NA	NA	NA	NA	NA	NA	NA	NA
NHL	−2.1 (−3.1 to −1.1)	<.001	1999 to 2002	−5.7 (−12.4 to 1.4)	.11	2002 to 2020	−1.5 (−2.0 to −1.0)	<.001	NA	NA	NA	NA	NA	NA
Leukemia	−0.9 (−1.5 to −0.3)	.004	1999 to 2020	−0.9 (−1.5 to −0.3)	.004	NA	NA	NA	NA	NA	NA	NA	NA	NA
**Women**														
Overall cancer	−1.1 (−1.2 to −1.0)	<.001	1999 to 2020	−1.1 (−1.2 to −1.0)	<.001	NA	NA	NA	NA	NA	NA	NA	NA	NA
Esophageal	−1.5 (−2.6 to −0.5)	.01	1999 to 2020	−1.5 (−2.6 to −0.5)	.01	NA	NA	NA	NA	NA	NA	NA	NA	NA
Stomach	−3.5 (−3.9 to −3.1)	<.001	1999 to 2020	−3.5 (−3.9 to −3.1)	<.001	NA	NA	NA	NA	NA	NA	NA	NA	NA
Colorectal	−1.8 (−2.2 to −1.4)	<.001	1999 to 2020	−1.8 (−2.2 to −1.4)	<.001	NA	NA	NA	NA	NA	NA	NA	NA	NA
Liver and intrahepatic bile duct	−1.5 (−2.0 to −1.0)	<.001	1999 to 2020	−1.5 (−2.0 to −1.0)	<.001	NA	NA	NA	NA	NA	NA	NA	NA	NA
Pancreatic	−0.2 (−1.5 to 1.1)	.77	1999 to 2008	−0.2 (−1.0 to 0.7)	.65	2008 to 2011	2.4 (−6.6 to 12.3)	.59	2011 to 2020	−1.1 (−1.9 to −0.2)	.02	NA	NA	NA
Lung and bronchial	−1.3 (−1.7 to −0.9)	<.001	1999 to 2013	−0.3 (−0.7 to 0.1)	.12	2013 to 2020	−3.2 (−4.4 to −2.0)	<.001	NA	NA	NA	NA	NA	NA
Myeloma	−1.5 (−2.4 to −0.6)	.003	1999 to 2020	−1.5 (−2.4 to −0.6)	.003	NA	NA	NA	NA	NA	NA	NA	NA	NA
Breast	−0.4 (−1.7 to 1.0)	.59	1999 to 2004	0.4 (−1.7 to 2.5)	.71	2004 to 2007	−3.8 (−12.5 to 5.7)	.39	2007 to 2020	0.2 (−0.3 to 0.7)	.50	NA	NA	NA
Cervical	−2.5 (−3.6 to −1.3)	<.001	1999 to 2009	−4.3 (−6.1 to −2.4)	<.001	2009 to 2020	−0.8 (−2.4 to 0.9)	.33	NA	NA	NA	NA	NA	NA
Uterine	2.5 (2.0 to 3.0)	<.001	1999 to 2020	2.5 (2.0 to 3.0)	<.001	NA	NA	NA	NA	NA	NA	NA	NA	NA
Ovarian	−0.8 (−1.1 to −0.4)	<.001	1999 to 2020	−0.8 (−1.1 to −0.4)	<.001	NA	NA	NA	NA	NA	NA	NA	NA	NA
Kidney and renal pelvic	−0.6 (−1.9 to 0.8)	.40	1999 to 2007	1.8 (−1.3 to 5.0)	.24	2007 to 2020	−2.0 (−3.4 to −0.6)	.01	NA	NA	NA	NA	NA	NA
Bladder	−0.9 (−1.7 to −0.2)	.02	1999 to 2020	−0.9 (−1.7 to −0.2)	.02	NA	NA	NA	NA	NA	NA	NA	NA	NA
Brain and CNS	1.4 (0.7 to 2.1)	<.001	1999 to 2020	1.4 (0.7 to 2.1)	<.001	NA	NA	NA	NA	NA	NA	NA	NA	NA
NHL	−2.3 (−2.7 to −1.8)	<.001	1999 to 2020	−2.3 (−2.7 to −1.8)	<.001	NA	NA	NA	NA	NA	NA	NA	NA	NA
Leukemia	−1.6 (−3.5 to 0.4)	.12	1999 to 2004	−4.2 (−7.7 to −0.5)	.03	2004 to 2011	2.7 (−0.1 to 5.7)	.06	2011 to 2015	−7.0 (−14.5 to 1.1)	.08	2015 to 2020	−0.4 (−4.0 to 3.4)	.83

Abbreviations: AAPC, average annual percent change; APC, annual percent change; CNS, central nervous system; NA, not applicable; NHL, non-Hodgkin lymphoma.

^a^ Segments were chosen by Joinpoint regression. Statistical significance was defined as *P* < .05. Significant values after Holm-Bonferroni correction are presented in eTable 3 in Supplement 1.

^b^ Year ranges across rows overlap per Joinpoint regression, representing inflection points where significant temporal trends occur.

**Figure 3.**
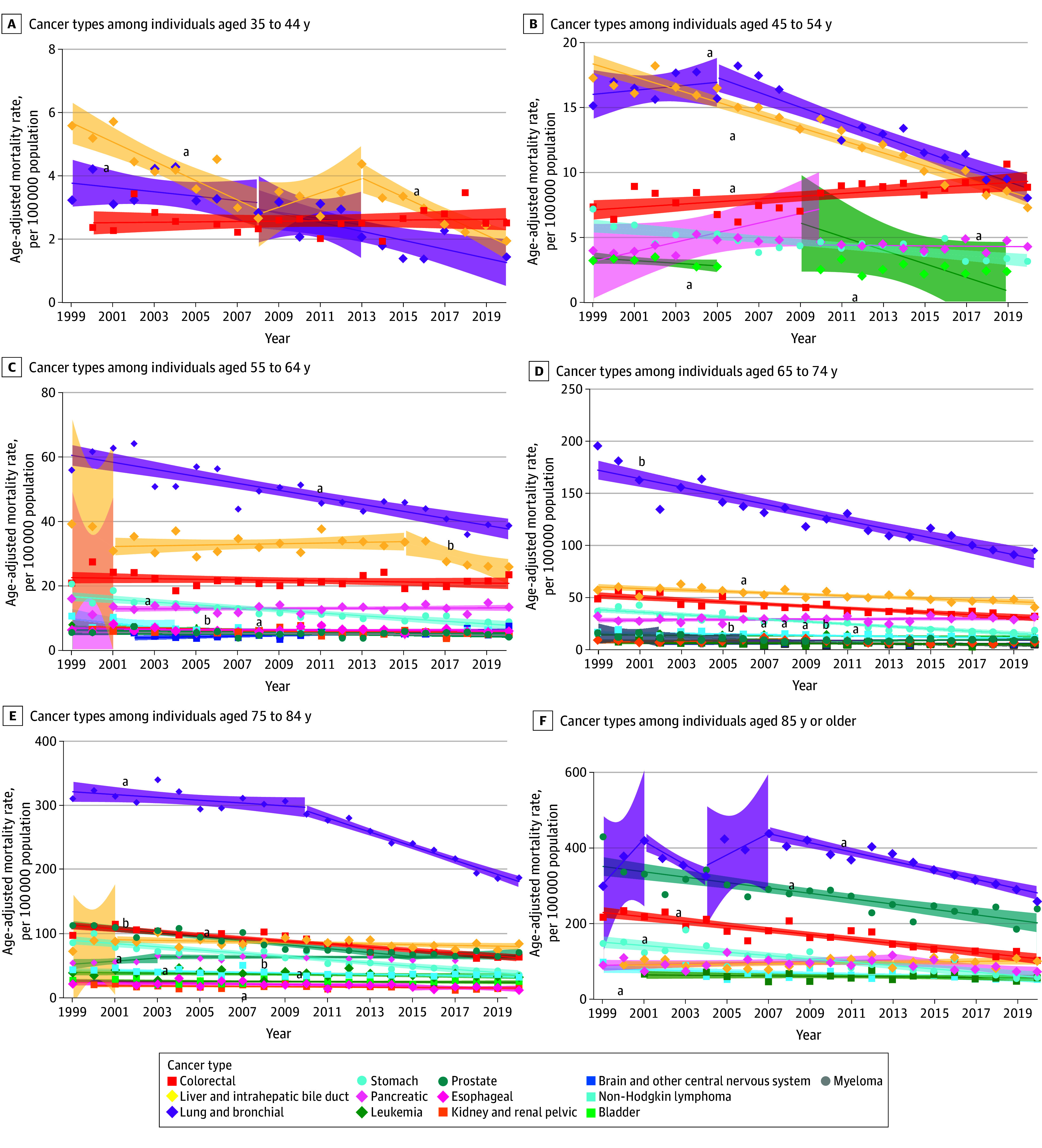
Trends in Age-Adjusted Mortality Rates Among Asian American and Pacific Islander Men by Age Group and Cancer Type, 1999-2020 Markers indicate observed rates; shading, 95% CIs; solid lines, modeled trends. ^a^Statistically significant average annual percent change after Holm-Bonferroni correction. ^b^*P* < .05, but not significant by Holm-Bonferroni correction.

**Figure 4.**
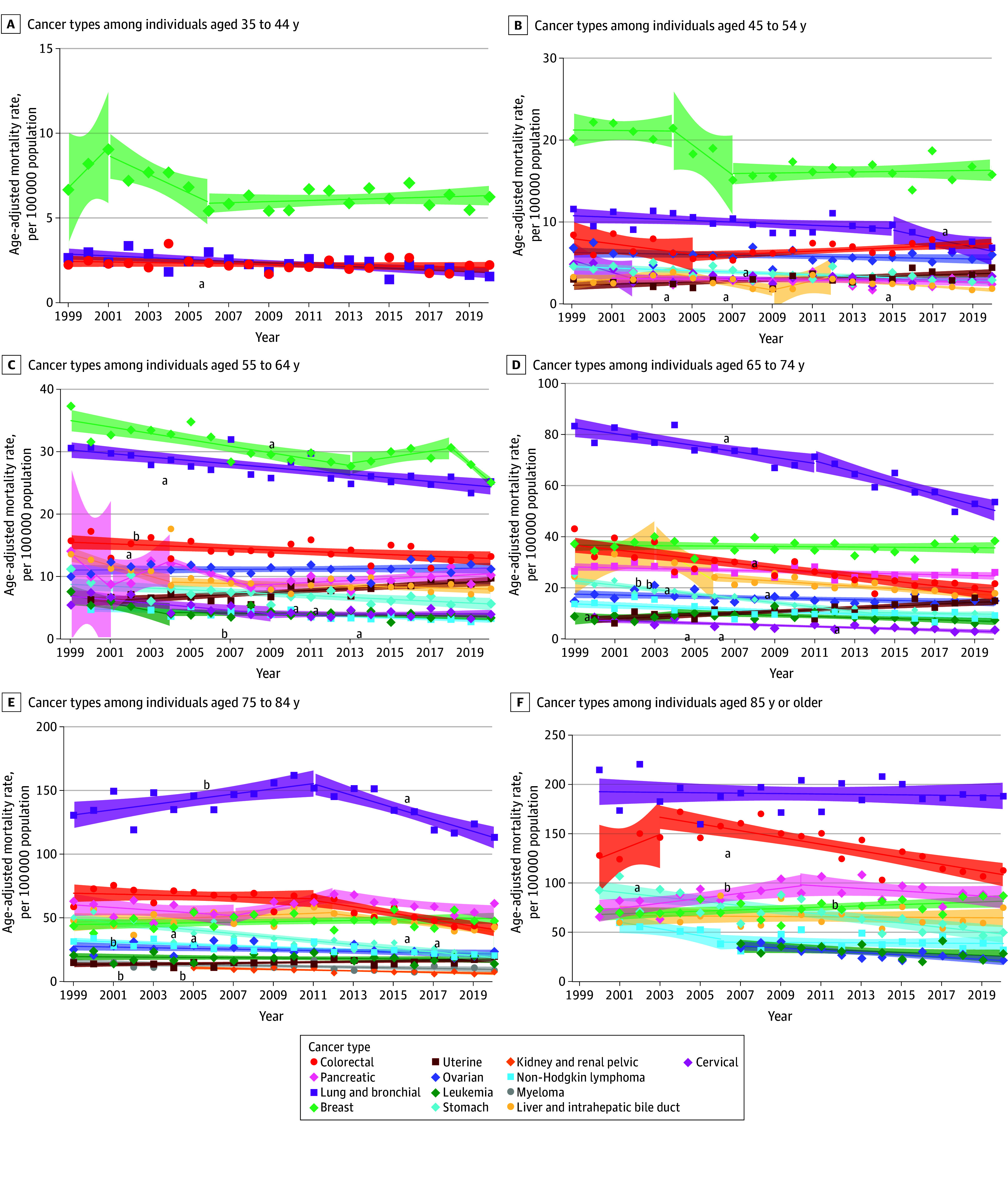
Trends in Age-Adjusted Mortality Rates Among Asian American and Pacific Islander Women by Age Group and Cancer Type, 1999-2020 Markers indicate observed rates; shading, 95% CIs; solid lines, modeled trends. ^a^Statistically significant average annual percent change after Holm-Bonferroni correction. ^b^*P* < .05, but not significant by Holm-Bonferroni correction.

By age group, CSM rates overall decreased across all ages (eTable 4 and eFigure 1 in Supplement 1; Figure 3 and Figure 4). The greatest decreases in mortality were seen among men aged 35 to 44 years for liver and intrahepatic bile duct cancer (AAPC, −5.0%; 95% CI, −7.7% to −2.2%; *P* < .001) and lung and bronchial cancer (AAPC, −4.8%; 95% CI, −6.3% to −3.2%; *P* < .001) (eTable 5 in Supplement 1). Colorectal cancer mortality significantly increased among men aged 45 to 54 years (AAPC, 1.3%; 95% CI, 0.5%-2.1%; *P* = .002) (eTable 5 in Supplement 1). Prostate cancer mortality rates among men aged 65 to 74 years initially decreased from 2002 to 2012 (2002-2006: APC, −2.7% [95% CI, −3.1% to −2.2%; *P* < .001]; 2006-2012: APC, −5.7% [95% CI, −10.9% to −0.2%; *P* = .04]), then increased from 2012 to 2020 (APC, 3.5%; 95% CI, 0.7%-6.4%; *P* = .02) (eTable 5 in Supplement 1). Uterine cancer mortality increased in women aged 45 to 54, 55 to 64, and 65 to 74 years (eTable 6 in Supplement 1), with the greatest increase in the 65- to 74-year age group (APC, 3.2%; 95% CI, 2.0%-4.4%; *P* = .001) (eTable 6 in Supplement 1).

By US census region, overall between 1999 and 2020, liver and intrahepatic bile duct cancer mortality increased for both men and women in all census regions (eTables 7 and 8; eFigures 2 and 3 in Supplement 1). Namely, CSM for liver and intrahepatic bile duct cancer increased in the Northeast (men: AAPC, 1.3% [95% CI, 0.9%-1.6%; *P* < .001]; women: AAPC, 1.5% [95% CI, 0.9%-2.1%; *P* < .001]), Midwest (men: AAPC, 1.8% [95% CI, 1.2%-2.4%; *P* < .001]; women: AAPC, 2.1% [95% CI, 1.8%-2.4%; *P* < .001]), South (men: AAPC, 2.1% [95% CI, 1.8%-2.3%; *P* < .001]; women: AAPC, 1.5% [95% CI, 1.1%-1.8%; *P* < .001]), and West (men: AAPC, 1.7% [95% CI, 1.3%-2.1%; *P* < .001]; women: AAPC, 1.9% [95% CI, 1.1%-2.7%; *P* < .001]).

Similarly, among women, uterine cancer mortality significantly increased from 1999 to 2020 in the Northeast (AAPC, 1.2%; 95% CI, 1.0%-1.4%; *P* < .001), Midwest (AAPC, 1.0%; 95% CI, 0.1%-1.9%; *P* = .03), South (AAPC, 1.0%; 95% CI, 0.5%-1.6%; *P* < .001), and West (AAPC, 1.3%; 95% CI, 0.9%-1.7%; *P* < .001). Pancreatic cancer mortality exhibited significant increases in the Midwest among men (AAPC, 0.6%; 95% CI, 0.5%-0.7%; *P* < .001) and women (AAPC, 0.4%; 95% CI, 0.3%-0.6%; *P* < .001) (eTables 7 and 8; eFigures 2 and 3 in Supplement 1).

## Discussion

In this cross-sectional study, overall, CSM rates among Asian American and Pacific Islander individuals decreased steadily from 1999 to 2020, particularly for lung and bronchial and stomach cancers. However, mortality rates increased for uterine and brain and CNS cancers among Asian American and Pacific Islander women during the same period. Additionally, liver and intrahepatic bile duct, uterine, and pancreatic cancer mortality rates increased significantly across most US census regions.

Decreases in mortality from certain cancers with infectious etiologies, such as stomach and cervical cancers, align with prior research indicating declining incidence rates.^9,10^ This trend in cervical cancer may be attributed to improvements in screening rates.^9,11^ The trend in stomach cancer may be due to improved hospital practices surrounding antibiotics against *Helicobacter pylori*, as well as dietary factors.^9,11^

Additionally, notable decreases in lung cancer deaths among Asian American and Pacific Islander men and women may be influenced by declining incidence rates of squamous cell carcinoma and small cell lung cancer among this population, as well as reductions in smoking rates among this subgroup and therapeutic advances.^9,12^ However, further research is warranted to understand lung cancer mortality trends between Asian American smokers and never-smokers. A recent study in California found that despite decreasing lung cancer incidence rates across all other racial and ethnic groups, rates increased among Asian American never-smokers by 2% annually, which is nearly double the incidence rates among never-smokers of other racial and ethnic groups.^13^ Thus, a significant proportion of Asian American women might not meet criteria for low-dose computed tomography screening per 2021 US Preventive Services Task Force guidelines, perpetuating lung cancer disparities. Furthermore, prior research revealed substantial variations in incidence and mortality attributed to tracheal, bronchial, and lung (TBL) cancers across Asian American and Pacific Islander ethnic groups. The incidence of TBL cancers has been reported to be disproportionately higher among Filipina and Korean women as well as Asian Indian and Pakistani men.^14^ In addition, TBL cancer deaths are considerably more common among Chinese, Korean, and Vietnamese populations, whereas these cancers are less common among Asian Indian and Japanese Americans.^2^ Such variations underscore the need for targeted risk reduction, screening, and treatment interventions tailored to the unique needs of diverse Asian American and Pacific Islander subpopulations.

Mortality rates increased for uterine cancer among Asian American and Pacific Islander women across all age groups, which may be associated with rising incidence rates alongside a higher prevalence of obesity, advanced-stage diagnoses, and aggressive histologic subtypes.^9,14^ Similarly, we found increasing mortality rates from CNS cancers among Asian American and Pacific Islander women from 1999 to 2020, contrary to prior research showing low incidence and high survival rates for gliomas,^15^ highlighting the need for additional study. Further research using disaggregated data is needed to elucidate specific disparities in CSM rates. For example, prior studies among Asian American and Pacific Islander subpopulations have shown that Asian Indian individuals have a relatively higher proportion of deaths from brain, meningeal, and other nervous system cancers, while Japanese and Korean American individuals have a lower proportion of these deaths.^2^

Increasing rates of colorectal cancer deaths observed among Asian American and Pacific Islander men aged 45 to 54 years appear to coincide with increased incidence rates in some Asian American and Pacific Islander subgroups, such as Korean men.^9,16^ This increased incidence may be influenced by elevated rates of smoking and alcohol use, as well as low screening rates, though further research using disaggregated Asian American data is warranted to illuminate disparities and identify targeted approaches.^9,16^ Similarly, an increasing incidence of liver cancer associated with HBV infection and alcohol consumption may contribute to the observed increase in liver cancer mortality across all US census regions.^9^

The model minority myth has praised the economic success of Asian American and Pacific Islander populations in a false comparison with other minority groups. The health care consequences of accepting this myth are that Asian American and Pacific Islander individuals could be assumed to have similar disease risk profiles to those of White individuals, obscuring substantial disparities in cancer mortality and other health outcomes across disaggregated Asian American and Pacific Islander subpopulations.^2^ This misconception neglects crucial variations in social, cultural, and linguistic factors (eg, English proficiency, health literacy levels, socioeconomic status, cultural perceptions of cancer, treatment-seeking behaviors) among specific Asian American and Pacific Islander ethnic groups that may erect barriers to accessing timely cancer screening, diagnosis, treatment, and support.^3^ These trends underscore the imperative for tailored, culturally relevant interventions to address cancer disparities among Asian American and Pacific Islander populations. For instance, empirical evidence has indicated that colorectal cancer mortality rates are notably higher among Korean American individuals. In response, community-based strategies have leveraged Korean church-based organizations to implement multifaceted programs that provide educational resources; facilitate care navigation; and expand access to screenings, such as the fecal immunochemical test, resulting in a remarkable increase in screening rates by more than 50% within the sample.^3^ Future research using disaggregated data must rigorously investigate the social and structural determinants that contribute to disproportionately higher incidence and mortality rates of specific cancer types in specific Asian American and Pacific Islander ethnic groups. Such research is essential for informing more targeted and effective interventions for cancer risk factor prevention and treatment.

To effectively address these national disparities in cancer mortality within Asian American and Pacific Islander populations, concerted efforts are needed by agencies such as the National Institutes of Health, CDC, and local health departments to enhance epidemiologic surveillance of cancer incidence and mortality among these communities. These initiatives should be conducted in collaboration with Asian American and Pacific Islander communities and organizations such as the Asian & Pacific Islander American Health Forum and the Asian American Network for Cancer Awareness, Research, and Training. The development of culturally tailored educational campaigns is essential. These interventions should account for diverse cultural beliefs and behaviors, including the use of traditional medicine, perceptions of cancer risk, familial decision-making processes, and more.^3,17^ For instance, a previous study showed that traditional Chinese medicine practitioners have played a crucial role in communicating culturally concordant information about cancer risk factors and prevention strategies for colorectal cancer, integrating traditional Chinese medicine concepts of healthy diets and positive emotion with biomedical information.^18^

Multisectoral partnerships are essential to addressing unique risk factors faced by specific Asian American and Pacific Islander ethnic groups, including linguistic barriers (eg, lack of English proficiency), socioeconomic disparities, and barriers to accessing primary care clinicians and cancer specialists.^3,12,19^ Further efforts are also needed to overcome the prevalent stigma surrounding cancer in many Asian American and Pacific Islander populations (eg, internalized shame about a cancer diagnosis or feeling like a burden to family and others), fostering open discussions and greater acceptance of preventive measures.^3,20^ Health care systems should integrate medical interpreters and care navigators fluent in specific languages and dialects of Asian American and Pacific Islander subgroups. Such navigators play a vital role in facilitating communication and building trust, helping to overcome linguistic barriers while providing culturally concordant care.^21,22^ These health care professionals ensure the accurate translation of evidence-based knowledge to Asian American and Pacific Islander patients, unlike family members or friends who often lack the expertise to navigate complex medical terminology surrounding cancer care.^3,21,22^ Health care professionals and policy makers should advocate for enhanced data systems that capture more detailed Asian American and Pacific Islander information on race, ethnicity, and other sociodemographic characteristics.^23^ They should also work to include Asian American and Pacific Islander individuals in clinical trials and national cancer registries to ensure their representation and address disparities in clinical research.

Additionally, health care professionals must receive training to recognize the unique genetic predispositions and environmental exposures prevalent among Asian American and Pacific Islander populations, such as higher rates of HBV infection, which is linked to liver cancer, in certain communities.^3^ Tailoring treatment plans to account for these factors might enhance diagnostic accuracy and therapeutic efficacy. Additional health system–level efforts are also needed to enhance cancer outcomes for Asian and Pacific Islander immigrants. Having a larger workforce of bilingual and bicultural health care professionals may build trust and improve understanding of cancer risks specific to different groups, with an emphasis on early detection.^3,24^ For instance, prior research has suggested that Vietnamese immigrants may exhibit a higher prevalence of risk factors, such as HBV infection, associated with their recent immigration from Vietnam compared with other Asian and Pacific Islander ethnic groups.^25^ Immigration histories have been found to be associated with cancer disparities.^3,24,25^ Korean and Vietnamese American individuals with recent immigration histories have higher incidence rates of cancers less prevalent in Western countries (eg, stomach and liver cancers), while Japanese and Filipino American individuals with longer immigration histories tend to experience higher incidence rates of cancers more commonly observed in the US, including colorectal and breast cancers.^24^ Moreover, immigration status may contribute to diagnosis trends and disease presentation. For example, research has indicated that the median age at prostate cancer diagnosis is higher among foreign-born Chinese, Japanese, Filipino, Korean, and Vietnamese men compared with US-born Japanese and Filipino men and US-born non-Hispanic Black and White men.^26^ Furthermore, foreign-born Chinese, Japanese, Filipino, Vietnamese, and non-Hispanic Black men may face a higher incidence of high-risk prostate cancer compared with their non-Hispanic White counterparts.^26^

### Limitations

This study is limited by its reliance on death certificates, which are prone to misclassification.^23^ Additionally, we did not include data on clinical factors, such as cancer stage and treatments, and social factors, including household income, educational attainment, employment status, health literacy, English proficiency, smoking status, insurance status, marital status, and US nativity. Future studies examining cancer mortality among Asian American and Pacific Islander populations should incorporate these crucial social and demographic factors to analyze more comprehensively their association with cancer mortality, particularly, their contributions to disparities across disaggregated Asian American and Pacific Islander ethnic groups. Studies examining temporal trends in the incidence of these cancer types are also needed. For instance, additional research is required to understand whether the increasing incidence of lung cancer among never-smoking Asian American and Pacific Islander women, as documented in previous studies,^13^ is associated with lung cancer mortality within this population.

Since CDC WONDER does not provide population totals or mortality rates for disaggregated Asian American and Pacific Islander subpopulations, this study relies on aggregated Asian American and Pacific Islander data. However, these aggregated analyses obscure critical health disparities among specific Asian American and Pacific Islander ethnic groups for certain cancer types. Additionally, this study offers a comprehensive overview of geographic trends across US census regions, but this approach may obscure critical variations at the state and county levels. Such granularity is imperative given the substantial differences in how state and local health departments conduct cancer epidemiologic surveillance, diagnosis, and treatment. These differences may be shaped by distinct health care policies, resource allocation, and community engagement strategies. Future research should therefore evaluate more specific geographic variations in cancer incidence and mortality among Asian American and Pacific Islander populations. Disaggregated data across various Asian American ethnic demographic characteristics, along with insights into cancer incidence and associated risk factors, are imperative to glean a nuanced context indispensable for formulating tailored screening strategies aimed at specific population subsets.

## Conclusions

In this cross-sectional study, we found that overall CSM rates decreased among Asian American and Pacific Islander individuals from 1999 to 2020, but specific cancer types exhibited increased mortality rates, with further disparities by sex, age, and US region. Targeted, culturally adapted clinical and public health interventions are imperative to narrowing disparities in cancer mortality.

## Notes

Supplement 1. **eTable 1.** Cancer Mortality by Type Based on *ICD-10* Codes **eTable 2.** Age-Adjusted Mortality Rates and Change in Mortality Rates per 100 000 Population for the Most Common Causes of Cancer Death by Sex Among Asian American and Pacific Islander Individuals in the US, 1999-2020 **eTable 3.** Annual Percent Changes in Cancer Death Rates by Sex and Cancer Type Among Asian American and Pacific Islander Individuals From 1999 to 2020 in the US With Holm-Bonferroni Correction **eTable 4.** Annual Percent Changes in Asian American and Pacific Islander Cancer Death Rates From 1999 to 2020 by Sex and Age Group **eTable 5.** Annual Percent Changes in Cancer Death Rates From 1999 to 2020 Among Asian American and Pacific Islander Men by Cancer Type and Age Group **eTable 6.** Annual Percent Changes in Cancer Death Rates From 1999 to 2020 Among Asian American and Pacific Islander Women by Cancer Type and Age Group **eTable 7.** Annual Percent Changes in Cancer Death Rates From 1999 to 2020 Among Asian American and Pacific Islander Men by Cancer Type and Census Region **eTable 8.** Annual Percent Changes in Cancer Death Rates From 1999 to 2020 Among Asian American and Pacific Islander Women by Cancer Type and Census Region **eFigure 1.** Trends in Asian American and Pacific Islander Age-Adjusted Cancer Death Rates From 1999-2020 by Sex and Age Group **eFigure 2.** Trends in Age-Adjusted Death Rates From 1999-2020 Among Asian American and Pacific Islander Men by Cancer Type and US Census Region **eFigure 3.** Trends in Age-Adjusted Death Rates From 1999-2020 Among Asian American and Pacific Islander Women by Cancer Type and US Census Region

Supplement 2.
Data Sharing Statement
