# Supplementary material for: US Cancer Mortality Trends Among Asian and Pacific Islander Populations
Source: JAMA Netw Open. 2024 Nov 4;7(11):e2442451. doi: 10.1001/jamanetworkopen.2024.42451 (PMC11581537; doi:10.1001/jamanetworkopen.2024.42451)
Supplement: Supplement 1. — eTable 1. Cancer Mortality by Type Based on ICD-10 Codes eTable 2. Age-Adjusted Mortality Rates and Change in Mortality Rates per 100 000 Population for the Most Common Causes of Cancer Death by Sex Among Asian American and Pacific Islander Individuals in the US, 1999-2020 eTable 3. Annual Percent Changes in Cancer Death Rates by Sex and Cancer Type Among Asian American and Pacific Islander Individuals From 1999 to 2020 in the US With Holm-Bonferroni Correction eTable 4. Annual Percent Changes in Asian American and Pacific Islander Cancer Death Rates From 1999 to 2020 by Sex and Age Group eTable 5. Annual Percent Changes in Cancer Death Rates From 1999 to 2020 Among Asian American and Pacific Islander Men by Cancer Type and Age Group eTable 6. Annual Percent Changes in Cancer Death Rates From 1999 to 2020 Among Asian American and Pacific Islander Women by Cancer Type and Age Group eTable 7. Annual Percent Changes in Cancer Death Rates From 1999 to 2020 Among Asian American and Pacific Islander Men by Cancer Type and Census Region eTable 8. Annual Percent Changes in Cancer Death Rates From 1999 to 2020 Among Asian American and Pacific Islander Women by Cancer Type and Census Region eFigure 1. Trends in Asian American and Pacific Islander Age-Adjusted Cancer Death Rates From 1999-2020 by Sex and Age Group eFigure 2. Trends in Age-Adjusted Death Rates From 1999-2020 Among Asian American and Pacific Islander Men by Cancer Type and US Census Region eFigure 3. Trends in Age-Adjusted Death Rates From 1999-2020 Among Asian American and Pacific Islander Women by Cancer Type and US Census Region [file jamanetwopen-e2442451-s001.pdf]

Supplemental Online Content

Zhu DT, Pompa IR, Qi D, Goldberg SI, Lee RJ, Kamran SC. US cancer mortality trends among Asian and Pacific Islander populations. *JAMA Netw Open*. 2024;7(11):e2442451. doi:10.1001/jamanetworkopen.2024.42451

eTable 1. Cancer Mortality by Type Based on *ICD-10* Codes

eTable 2. Age-Adjusted Mortality Rates and Change in Mortality Rates per 100 000 Population for the Most Common Causes of Cancer Death by Sex Among Asian American and Pacific Islander Individuals in the US, 1999-2020

eTable 3. Annual Percent Changes in Cancer Death Rates by Sex and Cancer Type Among Asian American and Pacific Islander Individuals From 1999 to 2020 in the US With Holm-Bonferroni Correction

eTable 4. Annual Percent Changes in Asian American and Pacific Islander Cancer Death Rates From 1999 to 2020 by Sex and Age Group

eTable 5. Annual Percent Changes in Cancer Death Rates From 1999 to 2020 Among Asian American and Pacific Islander Men by Cancer Type and Age Group

eTable 6. Annual Percent Changes in Cancer Death Rates From 1999 to 2020 Among Asian American and Pacific Islander Women by Cancer Type and Age Group

eTable 7. Annual Percent Changes in Cancer Death Rates From 1999 to 2020 Among Asian American and Pacific Islander Men by Cancer Type and Census Region

eTable 8. Annual Percent Changes in Cancer Death Rates From 1999 to 2020 Among Asian American and Pacific Islander Women by Cancer Type and Census Region

eFigure 1. Trends in Asian American and Pacific Islander Age-Adjusted Cancer Death Rates From 1999 to 2020 by Sex and Age Group

eFigure 2. Trends in Age-Adjusted Death Rates From 1999 to 2020 Among Asian American and Pacific Islander Men by Cancer Type and US Census Region

eFigure 3. Trends in Age-Adjusted Death Rates From 1999 to 2020 Among Asian American and Pacific Islander Women by Cancer Type and US Census Region

This supplemental material has been provided by the authors to give readers additional information about their work.

© 2024 Zhu DT et al. *JAMA Network Open*.

eTable 1. Cancer Mortality by Site Based on ICD-10 Codes

CDC WONDER Cancer Cause of Death	ICD-10 label
All Malignant Cancers	C00-C97
Bladder	C67
Brain and other Nervous System	C71, C72
Breast	C50
Cervix Uteri	C53
Colon and Rectum	C18-C20
Corpus and Uterus, part unspecified	C54-55
Esophagus	C15
Kidney and Renal Pelvis	C64-65
Leukemia	C91-95
Liver and Intrahepatic Bile	C22
Lung and Bronchus	C34
Myeloma	C90
Non-Hodgkin Lymphoma	C82, 83, 85
Ovary	C56
Pancreas	C25
Prostate	C61
Stomach	C16
Testis	C62

Abbreviations: ICD-10, International Classification of Diseases, Tenth Revision

© 2024 Zhu DT et al. *JAMA Network Open*.

eTable 2. Age-Adjusted Mortality Rates and Change in Mortality Rates per 100,000 Population for the Most Common Causes of Cancer Death by Sex Among Asian American and Pacific Islander Individuals in the US, 1999-2020

	**Men**			**Women**			**All Individuals**		
**Cancer Site or Type**	**Age-adjusted mortality rates**	**Deaths**	**Change in mortality rates**	**Age-adjusted mortality rates**	**Deaths**	**Change in mortality rates**	**Age-adjusted mortality rates**	**Deaths**	**Change in mortality rates**
**All Malignant Cancers**	123.8(123.2,124.5)	155936	-48.5 (-53.8 to -43.3)	89.3(88.9,89.8)	149450	-20.5 (-24.1,-17.0)	103.8(103.4,104.1)	305386	-31.2 (-34.3 to -28.2)
**Female Breast**	NA	NA	NA	11.8(11.7,12)	21154	-3.3 (-4.6 to -2.0)	6.6(6.5,6.7)	21292	-2.0 (-2.8 to -1.3)
**Cervix**	NA	NA	NA	1.9(1.8,1.9)	3391	-1.2 (-1.6 to -0.6)	1.0(1,1.1)	3391	-0.6 (-0.9 to -0.3)
**Colon and Rectum**	12.3(12.1,12.5)	15759	-4.8 (-6.5 to -3.1)	8.8(8.7,9.0)	14562	-3.6 (-4.8 to -2.4)	10.3(10.2,10.4)	30321	-3.9 (-4.9 to -3.0)
**Uterus**	NA	NA	NA	3.0(2.9,3.0)	5207	1.3 (0.6 to 1.8)	1.6(1.6,1.7)	5207	0.6 (0.2 to 0.9)
**Kidney and Renal Pelvis**	2.6(2.5,2.7)	3372	-0.5 (-1.5 to 0.4)	1.1(1.1,1.2)	1823	-0.5 (-1.0 to -0.1)	1.8(1.7,1.8)	5195	-0.5 (-1.0 to -0.1)
**Leukemia**	4.8(4.7,5.0)	6252	-1.3 (-2.2 to -0.2)	2.9(2.8,2.9)	4780	-1.3 (-1.9 to -0.6)	3.7(3.6,3.8)	11032	-1.2 (-1.8 to -0.7)
**Liver and Intrahepatic Bile Ducts**	14.0(13.8,14.2)	19143	2.2 (0.8 to 3.5)	5.9(5.8,6.0)	9574	0.9 (0.1 to 1.7)	9.5(9.4,9.6)	28717	1.6 (0.8 to 2.3)
**Lung and Bronchus**	31.5(31.1,31.8)	38927	-21.1 (-23.5 to -18.7)	17.3(17.1,17.5)	28286	-3.8 (-5.0 to -2.5)	23.3(23.2,23.5)	67213	-10.9 (-12.2 to -9.8)
**Non-Hodgkin Lymphoma**	4.7(4.6,4.8)	5730	-1.9 (-2.9 to -0.8)	3.0(2.9,3.1)	4840	-2.3 (-3.0 to -1.4)	3.7(3.6,3.8)	10570	-2.0 (-2.7 to -1.4)
**Ovary**	NA	NA	NA	4.6(4.5,4.7)	8087	-1.0 (-1.8 to -0.2)	2.6(2.5,2.6)	8087	-0.6 (-1.1 to -0.2)
**Pancreas**	8.2(8.1,8.4)	10304	0.5 (-0.7 to 1.8)	7.1(7.0,7.2)	11400	0.9 (-0.1 to 1.9)	7.6(7.5,7.7)	21704	0.8 (0.0 to 1.5)
**Prostate**	9.5(9.3,9.7)	9861	-8.8 (-10.8 to -6.6)	NA	NA	NA	3.8(3.8,3.9)	9861	-2.9 (-3.8 to -2.2)
**Stomach**	7.6(7.5,7.8)	9548	-3.8 (-5.0 to -2.7)	12.0(10.7,13.3)	7677	-1.5 (-2.2 to -0.7)	5.9(5.8,6)	17225	-2.6 (-3.2 to -1.8)
**Bladder**	2.8(2.7,2.9)	3129	-0.6 (-1.5 to 0.3)	0.9(0.9,1.0)	1418	0.1 (-0.4 to 0.4)	1.7(1.7,1.8)	4547	-0.2 (-0.6 to 0.3)

© 2024 Zhu DT et al. *JAMA Network Open*.

**Myeloma**	2.0(1.9,2.1)	2502	-0.3 (-1.0 to 0.4)	1.3(1.3,1.4)	2181	-0.9 (-1.5 to -0.3)	1.6(1.6,1.7)	4683	-0.7 (-1.2 to -0.3)
**Esophagus**	2.8(2.8,2.9)	3772	-1.4 (-2.3 to -0.6)	0.7(0.7,0.8)	1216	-0.4 (-0.7 to 0.0)	1.7(1.6,1.7)	4988	-0.7 (-1.1 to -0.4)
**Brain and other Central Nervous System**	2.5(2.5,2.6)	3677	0.4 (-0.2 to 1.1)	1.7(1.6,1.8)	2966	0.0 (-0.5 to 0.5)	2.1(2,2.1)	6643	0.2 (-0.2 to 0.6)

Rates are per 100,000 and age-adjusted to the 2000 US Std Population standard.

© 2024 Zhu DT et al. *JAMA Network Open*.

eTable 3. Annual Percent Changes in Cancer Death Rates by Sex and Cancer Type Among Asian American and Pacific Islander Individuals From 1999 to 2020 in the US With Holm-Bonferroni Correction

Cause of Death	Average APC from 1999-2020	p value	Segment 1	AAPC	p value	Segment 2	AAPC	p value	Segment 3	AAPC	p value	Segment 4	AAPC	p value
**All Malignant Cancers**	-1.5**(-1.7,-1.2)	<0.001	1999 - 2006	-1.6**(-1.9,-1.2)	<0.001	2006 - 2010	-0.5(-1.9,0.9)	0.47	2010 - 2020	-1.8**(-2.0,-1.5)	<0.001	NA	NA	NA
**Men**														
Overall Cancer	-1.8**(-2.2,-1.3)	<0.001	1999 - 2006	-1.8**(-2.2,-1.3)	<0.001	2006 - 2009	-0.3(-3.7,3.1)	0.83	2009 - 2020	-2.1**(-2.3,-1.9)	<0.001	NA	NA	NA
Esophagus	-1.3**(-2.0,-0.6)	0.001	1999 - 2020	-1.3**(-2.0,-0.6)	0.001	NA	NA	NA	NA	NA	NA	NA	NA	NA
Stomach	-3.8**(-4.1,-3.5)	<0.001	1999 - 2020	-3.8**(-4.1,-3.5)	<0.001	NA	NA	NA	NA	NA	NA	NA	NA	NA
Colon and Rectum	-1.6*(-3.1,-0.1)	0.04	1999 - 2001	6.4(-2.4,16.1)	0.14	2001 - 2005	-5.6*(-9.6,-1.4)	0.01	2005 - 2008	0.8(-7.5,10.0)	0.84	2008 - 2020	-2.2** (-2.7,-1.7)	<0.001
Liver and Intrahepatic Bile Ducts	-1.2(-2.4,0.0)	0.06	1999 - 2003	1.2(-1.3,3.8)	0.31	2003 - 2006	-3.9(-11.3,4.1)	0.29	2006 - 2011	1.0(-1.5,3.6)	0.41	2011 - 2020	-2.6** (-3.3,-1.9)	<0.001
Pancreas	0.0(-1.5,1.4)	0.95	1999 - 2001	-4.1(-12.4,5.0)	0.34	2001 - 2004	4.1(-4.9,14)	0.35	2004 - 2020	-0.3(-0.6,0.1)	0.09	NA	NA	NA
Lung and Bronchus	-2.6**(-2.9,-2.3)	<0.001	1999 - 2012	-1.5**(-1.9,-1.1)	<0.001	2012 - 2020	-4.3**(-5.1,-3.6)	<0.001	NA	NA	NA	NA	NA	NA
Myeloma	-0.8(-2.9,1.4)	0.50	1999 - 2002	-8.1(-17.8,2.6)	0.12	2002 - 2007	5.9(-1.2,13.6)	0.10	2007 - 2020	-1.5*(-2.6,-0.3)	0.02	NA	NA	NA
Kidney and Renal Pelvis	-1.0(-2.9,0.9)	0.30	1999 - 2004	-2.9(-7.3,1.8)	0.21	2004 - 2009	5.4(-1.4,12.7)	0.11	2009 - 2020	-3.0**(-4.3,-1.6)	<0.001	NA	NA	NA
Bladder	0.0(-0.7,0.6)	0.90	1999 - 2020	0.0(-0.7,0.6)	0.90	NA	NA	NA	NA	NA	NA	NA	NA	NA

© 2024 Zhu DT et al. *JAMA Network Open*.

Prostate	-1.9**(-2.8,-1.1)	<0.001	1999 - 2014	-2.8**(-3.5,-2.1)	<0.001	2014 - 2020	0.2(-2.6,3.0)	0.89	NA	NA	NA	NA	NA	NA
Brain and other Central Nervous System	0.7(0.0,1.5)	0.05	1999 - 2020	0.7(0.0,1.5)	0.05	NA	NA	NA	NA	NA	NA	NA	NA	NA
Non-Hodgkin Lymphoma	-2.1**(-3.1,-1.1)	<0.001	1999 - 2002	-5.7(-12.4,1.4)	0.11	2002 - 2020	-1.5**(-2.0,-1.0)	<0.001	NA	NA	NA	NA	NA	NA
Leukemia	-0.9**(-1.5,-0.3)	0.004	1999 - 2020	-0.9**(-1.5,-0.3)	0.004	NA	NA	NA	NA	NA	NA	NA	NA	NA
**Women**														
Overall Cancer	-1.1**(-1.2,-1.0)	<0.001	1999 - 2020	-1.1**(-1.2,-1.0)	<0.001	NA	NA	NA	NA	NA	NA	NA	NA	NA
Esophagus	-1.5**(-2.6,-0.5)	0.01	1999 - 2020	-1.5**(-2.6,-0.5)	0.01	NA	NA	NA	NA	NA	NA	NA	NA	NA
Stomach	-3.5**(-3.9,-3.1)	<0.001	1999 - 2020	-3.5**(-3.9,-3.1)	<0.001	NA	NA	NA	NA	NA	NA	NA	NA	NA
Colon and Rectum	-1.8**(-2.2,-1.4)	<0.001	1999 - 2020	-1.8**(-2.2,-1.4)	<0.001	NA	NA	NA	NA	NA	NA	NA	NA	NA
Liver and Intrahepatic Bile Ducts	-1.5**(-2.0,-1.0)	<0.001	1999 - 2020	-1.5**(-2.0,-1.0)	<0.001	NA	NA	NA	NA	NA	NA	NA	NA	NA
Pancreas	-0.2(-1.5,1.1)	0.77	1999 - 2008	-0.2(-1.0,0.7)	0.65	2008 - 2011	2.4(-6.6,12.3)	0.59	2011 - 2020	-1.1*(-1.9,-0.2)	0.02	NA	NA	NA
Lung and Bronchus	-1.3**(-1.7,-0.9)	<0.001	1999 - 2013	-0.3(-0.7,0.1)	0.12	2013 - 2020	-3.2**(-4.4,-2.0)	<0.001	NA	NA	NA	NA	NA	NA
Myeloma	-1.5**(-2.4,-0.6)	0.003	1999 - 2020	-1.5**(-2.4,-0.6)	0.003	NA	NA	NA	NA	NA	NA	NA	NA	NA

© 2024 Zhu DT et al. *JAMA Network Open*.

Breast	-0.4(-1.7,1.0)	0.59	1999 - 2004	0.4(-1.7,2.5)	0.71	2004 - 2007	-3.8(-12.5,5.7)	0.39	2007 - 2020	0.2(-0.3,0.7)	0.50	NA	NA	NA
Cervix	-2.5**(-3.6,-1.3)	<0.001	1999 - 2009	-4.3**(-6.1,-2.4)	<0.001	2009 - 2020	-0.8(-2.4,0.9)	0.33	NA	NA	NA	NA	NA	NA
Uterus	2.5**(2.0,3.0)	<0.001	1999 - 2020	2.5**(2.0, 3.0)	<0.001	NA	NA	NA	NA	NA	NA	NA	NA	NA
Ovary	-0.8**(-1.1,-0.4)	<0.001	1999 - 2020	-0.8**(-1.1,-0.4)	<0.001	NA	NA	NA	NA	NA	NA	NA	NA	NA
Kidney and Renal Pelvis	-0.6(-1.9,0.8)	0.40	1999 - 2007	1.8(-1.3,5.0)	0.24	2007 - 2020	-2.0*(-3.4,-0.6)	0.01	NA	NA	NA	NA	NA	NA
Bladder	-0.9*(-1.7,-0.2)	0.02	1999 - 2020	-0.9*(-1.7,-0.2)	0.02	NA	NA	NA	NA	NA	NA	NA	NA	NA
Brain and other Central Nervous System	1.4**(0.7,2.1)	<0.001	1999 - 2020	1.4**(0.7, 2.1)	<0.001	NA	NA	NA	NA	NA	NA	NA	NA	NA
Non-Hodgkin Lymphoma	-2.3**(-2.7,-1.8)	<0.001	1999 - 2020	-2.3**(-2.7,-1.8)	<0.001	NA	NA	NA	NA	NA	NA	NA	NA	NA
Leukemia	-1.6(-3.5,0.4)	0.12	1999 - 2004	-4.2*(-7.7,-0.5)	0.03	2004 - 2011	2.7(-0.1,5.7)	0.06	2011 - 2015	-7.0(-14.5,1.1)	0.08	-0.3812 (-4.0393…3.4163)	-0.4(-4.0,3.4)	0.83

Segments were chosen by Joinpoint regression.
Statistically significant values in red. ** indicates the p-value is significant after Holm-Bonferroni correction. * indicates the p-value is <0.05, but not significant after Holm-Bonferroni correction.
Abbreviations: APC, Annual percentage change; NA, not applicable

© 2024 Zhu DT et al. *JAMA Network Open*.

eTable 4. Annual Percent Changes in Asian American and Pacific Islander Cancer Death Rates From 1999 to 2020 by Sex and Age Group

Age Group	Average APC from 1999-2020 (95%CI)	p value	Segment 1		p value	Segment 2		p value	Segment 3		p value
**Men**											
15-24 years old	-2.5(-5.8,0.9)	0.14	1999 - 2001	-20.5(-43.2,11.4)	0.17	2001 - 2012	1.9(-0.7,4.6)	0.14	2012 - 2020	-3.6(-7.1,0.0)	0.05
25-34 years old	-1.5**(-2.3,-0.8)	<0.001	1999 - 2020	-1.5**(-2.3,-0.8)	<0.001	NA	NA	NA	NA	NA	NA
35-44 years old	-2.2**(-2.7,-1.7)	<0.001	1999 - 2020	-2.2**(-2.7,-1.7)	<0.001	NA	NA	NA	NA	NA	NA
45-54 years old	-2.1**(-2.8,-1.4)	<0.001	1999 - 2017	-1.7**(-2.0,-1.3)	<0.001	2017 - 2020	-4.7(-9.4,0.1)	0.06	NA	NA	NA
55-64 years old	-1.2(-2.4,0.0)	0.05	1999 - 2001	0.6(-6.7,8.4)	0.87	2001 - 2004	-5.1(-11.9,2.3)	0.16	2004 - 2020	-0.7**(-1.0,-0.4)	<0.001
65-74 years old	-2.1**(-2.7,-1.5)	<0.001	1999 - 2002	-4.8*(-8.9,-0.4)	0.03	2002 - 2020	-1.7**(-1.9,-1.4)	<0.001	NA	NA	NA
75-84 years old	-1.7**(-2.1,-1.4)	<0.001	1999 - 2008	-0.8*(-1.4,-0.1)	0.03	2008 - 2020	-2.4**(-2.9,-2.0)	<0.001	NA	NA	NA
85+ years old	-1.8**(-2.1,-1.4)	<0.001	1999 - 2002	-3.1**(-5.3,-1.0)	0.01	2002 - 2009	0.3(-0.4,1.1)	0.34	2009 - 2020	-2.7**(-3.0,-2.4)	<0.001
**Women**											
15-24 years old	-1.4*(-2.4,-0.3)	0.02	2000 - 2020	-1.4*(-2.4,-0.3)	0.02	NA	NA	NA	NA	NA	NA

© 2024 Zhu DT et al. *JAMA Network Open*.

25-34 years old	-1.3**(-2.1,-0.6)	0.001	1999 - 2020	-1.3**(-2.1,-0.6)	0.001	NA	NA	NA	NA	NA	NA
35-44 years old	-2.0**(-3.2,-0.9)	0.001	1999 - 2009	-2.4**(-3.4,-1.3)	<0.001	2009 - 2016	0.8(-1.6,3.2)	0.50	2016 - 2020	-5.9**(-10.0,-1.6)	0.01
45-54 years old	-1.1**(-1.5,-0.8)	<0.001	1999 - 2020	-1.1**(-1.5,-0.8)	<0.001	NA	NA	NA	NA	NA	NA
55-64 years old	-1.0**(-1.5,-0.5)	<0.001	1999 - 2008	-2.0**(-2.9,-1.1)	<0.001	2008 - 2020	-0.2(-0.8,0.4)	0.48	NA	NA	NA
65-74 years old	-1.4**(-2.0,-0.9)	<0.001	1999 - 2017	-1.9**(-2.1,-1.6)	<0.001	2017 - 2020	1.2(-2.8,5.3)	0.55	NA	NA	NA
75-84 years old	-1.2**(-1.6,-0.8)	<0.001	1999 - 2004	-1.4*(-2.6,-0.2)	0.02	2004 - 2011	0.8(-0.1,1.7)	0.09	2011 - 2020	-2.6**(-3.1,-2.1)	<0.001
85+ years old	-0.5*(-0.9,0.0)	0.03	1999 - 2008	0.7(-0.1,1.6)	0.07	2008 - 2020	-1.4**(-1.9,-0.8)	<0.001	NA	NA	NA

Segments were chosen by Joinpoint regression.
Statistically significant values in red. ** indicates the p-value is significant after Holm-Bonferroni correction. * indicates the p-value is <0.05, but not significant after Holm-Bonferroni correction.
Abbreviations: APC, Annual percentage change; NA, not applicable

© 2024 Zhu DT et al. *JAMA Network Open*.

eTable 5. Annual Percent Changes in Cancer Death Rates From 1999 to 2020 Among Asian American and Pacific Islander Men by Cancer Type and Age Group

Cause of Death	Average APC from 1999-2020 (95%CI)	p value	Segment 1		p value	Segment 2		p value	Segment 3		p value	Segment 4		p value
**Men**														
**35-44 years old**														
Colon and Rectum	0.2(-0.9,1.4)	0.69	2000-2020	0.2(-0.9,1.4)	0.69	NA	NA	NA	NA	NA	NA	NA	NA	NA
Liver and Intrahepatic Bile Ducts	-5.0**(-7.7,-2.2)	<0.001	1999-2008	-7.2**(-9.9,-4.3)	<0.001	NA	NA	NA	2013-2020	-8.7**(-12.6,-4.6)	0.001	NA	NA	NA
Lung and Bronchus	-4.8**(-6.3,-3.2)	<0.001	1999-2020	-4.8**(-6.3,-3.2)	<0.001	2008-2013	4.8(-5.5,16.2)	0.35	NA	NA	NA	NA	NA	NA
**45-54 years old**														
Stomach	-2.7**(-3.5,-1.9)	<0.001	1999-2020	-2.7**(-3.5,-1.9)	<0.001	NA	NA	NA	NA	NA	NA	NA	NA	NA
Colon and Rectum	1.3**(0.5,2.1)	0.002	1999-2020	1.3**(0.5,2.1)	0.002	NA	NA	NA	NA	NA	NA	NA	NA	NA
Liver and Intrahepatic Bile Ducts	-3.9**(-4.7,-3.2)	<0.001	1999-2010	-2.1**(-3.2,-1.1)	<0.001	NA	NA	NA	NA	NA	NA	NA	NA	NA
Pancreas	0.5(-1.0,2.1)	0.51	1999-2005	3.8(-1.2,9.0)	0.13	2010-2020	-5.8**(-7.0,-4.6)	<0.001	NA	NA	NA	NA	NA	NA
Lung and Bronchus	-2.7**(-3.8,-1.6)	<0.001	1999-2006	1.4(-1.6,4.5)	0.34	2005-2020	-0.8(-2,0.5)	0.21	NA	NA	NA	NA	NA	NA
Leukemia	-1.7**(-2.7,-0.8)	0.001	1999-2019	-1.7**(-2.7,-0.8)	0.001	2006-2020	-4.7**(-5.7,-3.7)	<0.001	NA	NA	NA	NA	NA	NA
**55-64 years old**														

© 2024 Zhu DT et al. *JAMA Network Open*.

Stomach	-3.4**(-4.1,-2.6)	<0.001	1999-2020	-3.4**(-4.1,-2.6)	<0.001	NA	NA	NA	NA	NA	NA	NA	NA	NA
Colon and Rectum	-0.4(-1.0,0.3)	0.27	1999-2020	-0.4(-1.0,0.3)	0.27	NA	NA	NA	NA	NA	NA	NA	NA	NA
Liver and Intrahepatic Bile Ducts	-2.3(-4.8,0.2)	0.07	1999-2001	-10.6(-30.1,14.4)	0.35	NA	NA	NA	2015-2020	-6.0*(-11.0,-0.7)	0.03	NA	NA	NA
Pancreas	0.0(-0.7,0.7)	0.99	1999-2020	0.0(-0.7,0.7)	0.99	2001-2015	0.3(-1,1.6)	0.67	NA	NA	NA	NA	NA	NA
Lung and Bronchus	-2.2**(-2.7,-1.7)	<0.001	1999-2020	-2.2**(-2.7,-1.7)	<0.001	NA	NA	NA	NA	NA	NA	NA	NA	NA
Prostate	-0.8(-2.2,0.6)	0.22	1999-2020	-0.8(-2.2,0.6)	0.22	NA	NA	NA	NA	NA	NA	NA	NA	NA
Esophagus	-0.4(-1.2,0.5)	0.38	1999-2020	-0.4(-1.2,0.5)	0.38	NA	NA	NA	NA	NA	NA	NA	NA	NA
Kidney and Renal Pelvis	-1.4*(-2.7,-0.1)	0.04	2000-2020	-1.4*(-2.7,-0.1)	0.04	NA	NA	NA	NA	NA	NA	NA	NA	NA
Brain and other Central Nervous System	0.9(-3.5,5.4)	0.70	2002-2004	-17.4(-45.5,25.3)	0.34	NA	NA	NA	NA	NA	NA	NA	NA	NA
Non-Hodgkin Lymphoma	-2.6*(-4.8,-0.4)	0.02	1999-2005	-7.1(-13.6,-0.2)	0.05	2004-2020	3.4**(1.8,5.1)	<0.001	NA	NA	NA	NA	NA	NA
Leukemia	-1.2(-2.5,0.2)	0.08	1999-2020	-1.2(-2.5,0.2)	0.08	2005-2020	-0.8(-2.6,1)	0.37	NA	NA	NA	NA	NA	NA
**65-74 years old**														
Stomach	-4.0**(-4.5,-3.4)	<0.001	1999-2020	-4.0**(-4.5,-3.4)	<0.001	NA	NA	NA	NA	NA	NA	NA	NA	NA

© 2024 Zhu DT et al. *JAMA Network Open*.

Colon and Rectum	-2.3**(-2.8,-1.8)	<0.001	1999-2020	-2.3**(-2.8,-1.8)	<0.001	NA	NA	NA	NA	NA	NA	NA	NA	NA
Liver and Intrahepatic Bile Ducts	-1.3**(-1.7,-0.9)	<0.001	1999-2020	-1.3**(-1.7,-0.9)	<0.001	NA	NA	NA	NA	NA	NA	NA	NA	NA
Pancreas	0.3(-0.3,0.9)	0.30	1999-2020	0.3(-0.3,0.9)	0.30	NA	NA	NA	NA	NA	NA	NA	NA	NA
Lung and Bronchus	-3.4**(-4.4,-2.4)	<0.001	1999-2002	-7.5*(-13.9,-0.6)	0.04	NA	NA	NA	NA	NA	NA	NA	NA	NA
Prostate	-0.6(-3.7,2.6)	0.71	1999-2002	-9.7(-20.5,2.4)	0.10	2002-2006	-2.7**(-3.1,-2.2)	<0.001	2006-2012	-5.7*(-10.9,-0.2)	0.04	2012-2020	3.5**(0.7,6.4)	0.02
Esophagus	-2.0**(-3.4,-0.6)	0.01	1999-2008	-5.8**(-8.3,-3.2)	<0.001	2002-2006	6.7(-6.0,21.1)	0.28	NA	NA	NA	NA	NA	NA
Kidney and Renal Pelvis	-1.1*(-2.1,0.0)	0.04	1999-2020	-1.1*(-2.1,0.0)	0.04	2008-2020	1(-0.8,2.8)	0.26	NA	NA	NA	NA	NA	NA
Bladder	-1.0(-2.4,0.4)	0.14	2000-2020	-1.0(-2.4,0.4)	0.14	NA	NA	NA	NA	NA	NA	NA	NA	NA
Brain and other Central Nervous System	-0.2(-1.9,1.4)	0.75	2002-2020	-0.2(-1.9,1.4)	0.75	NA	NA	NA	NA	NA	NA	NA	NA	NA
Non-Hodgkin Lymphoma	-1.6**(-2.4,-0.9)	<0.001	1999-2020	-1.6**(-2.4,-0.9)	<0.001	NA	NA	NA	NA	NA	NA	NA	NA	NA
Leukemia	-0.8*(-1.6,0.0)	0.04	1999-2020	-0.8*(-1.6,0.0)	0.04	NA	NA	NA	NA	NA	NA	NA	NA	NA
Myeloma	-2.2**(-3.4,-0.9)	0.002	1999-2020	-2.2**(-3.4,-0.9)	0.002	NA	NA	NA	NA	NA	NA	NA	NA	NA
**75-84 years old**														

© 2024 Zhu DT et al. *JAMA Network Open*.

Stomach	-4.0**(-4.7,-3.3)	<0.001	1999-2020	-4.0**(-4.7,-3.3)	<0.001	NA	NA	NA	NA	NA	NA	NA	NA	NA
Colon and Rectum	-2.2*(-3.8,-0.5)	0.01	1999-2001	6.8(-11.3,28.5)	0.46	NA	NA	NA	NA	NA	NA	NA	NA	NA
Liver and Intrahepatic Bile Ducts	0.3(-1.6,2.2)	0.77	1999-2001	10.1(-10.2,35)	0.33	2001-2020	-3.1**(-3.6,-2.5)	<0.001	NA	NA	NA	NA	NA	NA
Pancreas	0.8(-0.2,1.9)	0.13	1999-2004	3.6(-0.7,8.1)	0.10	2001-2020	-0.7*(-1.3,-0.1)	0.02	NA	NA	NA	NA	NA	NA
Lung and Bronchus	-2.6**(-3.1,-2.1)	<0.001	1999-2010	-0.6(-1.4,0.1)	0.08	2004-2020	0(-0.7,0.7)	0.95	NA	NA	NA	NA	NA	NA
Prostate	-2.4**(-3.5,-1.3)	<0.001	1999-2015	-3.4**(-4.1,-2.7)	<0.001	2010-2020	-4.7**(-5.5,-3.9)	<0.001	NA	NA	NA	NA	NA	NA
Esophagus	-2.4**(-3.7,-1.1)	0.002	1999-2020	-2.4**(-3.7,-1.1)	0.002	2015-2020	0.9(-3.4,5.3)	0.67	NA	NA	NA	NA	NA	NA
Kidney and Renal Pelvis	-1.1(-2.2,0.1)	0.06	2000-2020	-1.1(-2.2,0.1)	0.06	NA	NA	NA	NA	NA	NA	NA	NA	NA
Bladder	-0.3(-1.3,0.6)	0.47	1999-2020	-0.3(-1.3,0.6)	0.47	NA	NA	NA	NA	NA	NA	NA	NA	NA
Non-Hodgkin Lymphoma	-1.3**(-1.9,-0.7)	<0.001	1999-2020	-1.3**(-1.9,-0.7)	<0.001	NA	NA	NA	NA	NA	NA	NA	NA	NA
Leukemia	-0.7(-1.7,0.3)	0.18	1999-2020	-0.7(-1.7,0.3)	0.18	NA	NA	NA	NA	NA	NA	NA	NA	NA
**>85 years old**														
Stomach	-4.1**(-5.2,-3.1)	<0.001	1999-2020	-4.1**(-5.2,-3.1)	<0.001	NA	NA	NA	NA	NA	NA	NA	NA	NA
Colon and Rectum	-3.4**(-4.1,-2.8)	<0.001	1999-2020	-3.4**(-4.1,-2.8)	<0.001	NA	NA	NA	NA	NA	NA	NA	NA	NA

© 2024 Zhu DT et al. *JAMA Network Open*.

Liver and Intrahepatic Bile Ducts	0.6(-0.3,1.5)	0.19	2000-2020	0.6(-0.3,1.5)	0.19	NA	NA	NA	NA	NA	NA	NA	NA	NA
Pancreas	-1.0(-2.9,0.9)	0.28	1999-2013	0.9(-0.8,2.7)	0.28	NA	NA	NA	NA	NA	NA	NA	NA	NA
Lung and Bronchus	-0.4(-3.4,2.8)	0.81	1999-2001	16.6(-0.1,36.1)	0.05	2013-2020	-4.8(-9.4,0.1)	0.05	2004-2007	9.3(-6.3,27.5)	0.23	2007-2020	-3.5**(-4.2,-2.7)	<0.001
Prostate	-2.4**(-3.1,-1.8)	<0.001	1999-2020	-2.4**(-3.1,-1.8)	<0.001	2001-2004	-6.3(-19.7,9.4)	0.37	NA	NA	NA	NA	NA	NA
Bladder	-0.6(-2.0,0.7)	0.33	2001-2020	-0.6(-2.0,0.7)	0.33	NA	NA	NA	NA	NA	NA	NA	NA	NA
Non-Hodgkin Lymphoma	-1.7**(-2.8,-0.6)	0.01	1999-2020	-1.7**(-2.8,-0.6)	0.01	NA	NA	NA	NA	NA	NA	NA	NA	NA

Segments were chosen by Joinpoint regression.
Statistically significant values in red. ** indicates the p-value is significant after Holm-Bonferroni correction. * indicates the p-value is <0.05, but not significant after Holm-Bonferroni correction.
Abbreviations: APC, Annual percentage change; NA, not applicable

© 2024 Zhu DT et al. *JAMA Network Open*.

eTable 6. Annual Percent Changes in Cancer Death Rates From 1999 to 2020 Among Asian American and Pacific Islander Women by Cancer Type and Age Group

Cause of Death	Average APC from 1999-2020 (95%CI)	p value	Segment 1		p value	Segment 2		p value	Segment 3		p value	Segment 4		p value
**Women**														
**35-44 years**														
Colon and Rectum	-0.8(-1.9,0.3)	0.16	1999-2020	-0.8(-1.9,0.3)	0.16	NA	NA	NA	NA	NA	NA	NA	NA	NA
Lung and Bronchus	-2.4**(-3.6,-1.1)	<0.001	1999-2020	-2.4**(-3.6,-1.1)	<0.001	NA	NA	NA	NA	NA	NA	NA	NA	NA
Breast	-0.4(-3.4,2.6)	0.78	1999-2001	14.1(-12.3,48.4)	0.30	2001-2006	-7.7(-15.1,0.3)	0.06	2006-2020	0.3(-0.9,1.6)	0.57	NA	NA	NA
**45-54 years**														
Colon and Rectum	-0.4(-2.2,1.4)	0.67	1999-2005	-4.7(-10,0.9)	0.10	2005-2020	1.4(-0.1,2.9)	0.06	NA	NA	NA	NA	NA	NA
Pancreas	-0.9(-2.6,0.8)	0.28	2001-2020	-0.9(-2.6,0.8)	0.28	NA	NA	NA	NA	NA	NA	NA	NA	NA
Lung and Bronchus	-2.4**(-3.9,-0.8)	0.003	1999-2015	-1.1(-2.2,0)	0.05	2015-2020	-6.4*(-12.2,-0.2)	0.04	NA	NA	NA	NA	NA	NA
Breast	-1.3(-4.2,1.8)	0.41	1999-2004	-0.2(-4.8,4.7)	0.93	2004-2007	-8.6(-26.1,13.1)	0.38	2007-2020	0.1(-1,1.2)	0.89	NA	NA	NA
Stomach	-2.2**(-3.2,-1.2)	<0.001	1999-2020	-2.2**(-3.2,-1.2)	<0.001	NA	NA	NA	NA	NA	NA	NA	NA	NA
Liver and Intrahepatic Bile Ducts	-2.1(-7.8,4)	0.49	1999-2004	6(-2.8,15.5)	0.17	2004-2009	-12.7*(-22.7,-1.4)	0.03	2009-2012	19.2(-18.9,75.3)	0.34	2012-2020	-7**(-10.8,-3.0)	0.003
Cervix	-2.9(-5.8,0)	0.05	1999-2002	-15.5(-31.6,4.4)	0.11	2002-2020	-0.6(-2,0.7)	0.33	NA	NA	NA	NA	NA	NA

© 2024 Zhu DT et al. *JAMA Network Open*.

Uterus	2.2**(0.8,3.7)	0.01	1999-2020	2.2**(0.8,3.7)	0.01	NA	NA	NA	NA	NA	NA	NA	NA	NA
Ovary	-0.6(-1.5,0.3)	0.17	1999-2020	-0.6(-1.5,0.3)	0.17	NA	NA	NA	NA	NA	NA	NA	NA	NA
**55-64 years**														
Colon and Rectum	-0.9*(-1.5,-0.2)	0.01	1999-2020	-0.9*(-1.5,-0.2)	0.01	NA	NA	NA	NA	NA	NA	NA	NA	NA
Pancreas	-1.3(-7,4.8)	0.68	1999-2001	-21.9(-44.3,9.6)	0.14	2001-2004	15.5(-17.6,62)	0.37	2004-2008	-9.7(-23.7,7)	0.21	2008-2020	1.7(-0.3,3.8)	0.09
Lung and Bronchus	-1.0**(-1.4,-0.7)	<0.001	1999-2020	-1.0**(-1.4,-0.7)	<0.001	NA	NA	NA	NA	NA	NA	NA	NA	NA
Breast	-1.6(-3.3,0.2)	0.07	1999-2013	-1.6**(-2.3,-0.9)	<0.001	2013-2018	1.9(-2.9,6.8)	0.42	2018-2020	-9.5(-22.2,5.2)	0.18	NA	NA	NA
Stomach	-2.0**(-3.0,-0.9)	<0.001	1999-2020	-2.0**(-3.0,-0.9)	<0.001	NA	NA	NA	NA	NA	NA	NA	NA	NA
Liver and Intrahepatic Bile Ducts	-2.6**(-4,-1.2)	<0.001	1999-2004	-7.7**(-12.9,-2.2)	0.01	2004-2020	-1(-1.9,0)	0.06	NA	NA	NA	NA	NA	NA
Cervix	-2.7*(-4.9,-0.4)	0.02	1999-2009	-5.4*(-8.9,-1.8)	0.01	2009-2020	-0.1(-3.4,3.2)	0.93	NA	NA	NA	NA	NA	NA
Uterus	2.1**(1.3,3)	<0.001	2000-2020	2.1**(1.3,3)	<0.001	NA	NA	NA	NA	NA	NA	NA	NA	NA
Ovary	0.1(-0.5,0.8)	0.73	1999-2020	0.1(-0.5,0.8)	0.73	NA	NA	NA	NA	NA	NA	NA	NA	NA
Leukemia	-3.2(-6.6,0.3)	0.07	1999-2004	-9.7(-22.4,5.1)	0.17	2004-2020	-1.1(-2.9,0.7)	0.21	NA	NA	NA	NA	NA	NA
Non-Hodgkin Lymphoma	-2.4**(-3.4,-1.5)	<0.001	2000-2020	-2.4**(-3.4,-1.5)	<0.001	NA	NA	NA	NA	NA	NA	NA	NA	NA

© 2024 Zhu DT et al. *JAMA Network Open*.

**65-74 years**														
Colon and Rectum	-3.1**(-3.8,-2.3)	<0.001	1999-2020	-3.1**(-3.8,-2.3)	<0.001	NA	NA	NA	NA	NA	NA	NA	NA	NA
Pancreas	-0.6*(-1.1,-0.2)	0.01	1999-2020	-0.6*(-1.1,-0.2)	0.01	NA	NA	NA	NA	NA	NA	NA	NA	NA
Lung and Bronchus	-2.3**(-2.9,-1.6)	<0.001	1999-2011	-1.5**(-2.3,-0.7)	0.001	2011-2020	-3.3**(-4.5,-2)	<0.001	NA	NA	NA	NA	NA	NA
Breast	-0.1(-0.6,0.4)	0.57	1999-2020	-0.1(-0.6,0.4)	0.57	NA	NA	NA	NA	NA	NA	NA	NA	NA
Stomach	-4.1**(-5.2,-3.1)	<0.001	1999-2014	-5.1**(-5.9,-4.2)	<0.001	2014-2020	-1.8(-5.2,1.8)	0.30	NA	NA	NA	NA	NA	NA
Liver and Intrahepatic Bile Ducts	-2.1(-5.8,1.8)	0.28	1999-2003	7.3(-1.5,16.7)	0.10	2003-2006	-12.4(-33,14.5)	0.31	2006-2020	-2.3**(-3.6,-1.1)	0.001	NA	NA	NA
Cervix	-4.1**(-5.4,-2.9)	<0.001	2000-2020	-4.1**(-5.4,-2.9)	<0.001	NA	NA	NA	NA	NA	NA	NA	NA	NA
Uterus	3.2**(2.0,4.4)	<0.001	2000-2020	3.2**(2.0,4.4)	<0.001	NA	NA	NA	NA	NA	NA	NA	NA	NA
Ovary	-0.9*(-1.7,-0.2)	0.02	1999-2020	-0.9*(-1.7,-0.2)	0.02	NA	NA	NA	NA	NA	NA	NA	NA	NA
Leukemia	-0.9(-2.7,0.9)	0.32	1999-2009	2.6(-0.3,5.7)	0.08	2009-2020	-4.0**(-6.4,-1.6)	0.003	NA	NA	NA	NA	NA	NA
Non-Hodgkin Lymphoma	-2.5**(-3.6,-1.4)	<0.001	1999-2020	-2.5**(-3.6,-1.4)	<0.001	NA	NA	NA	NA	NA	NA	NA	NA	NA
**75-84 years**														
Colon and Rectum	-2.4**(-3.6,-1.2)	<0.001	1999-2011	-0.7(-2.1,0.9)	0.37	2011-2020	-4.7**(-6.9,-2.4)	<0.001	NA	NA	NA	NA	NA	NA

© 2024 Zhu DT et al. *JAMA Network Open*.

Pancreas	-0.4(-2.9,2)	0.72	1999-2007	-1.7(-4.7,1.3)	0.24	2007-2012	5.3(-3.7,15.1)	0.24	2012-2020	-2.6(-5.5,0.5)	0.09	NA	NA	NA
Lung and Bronchus	-0.7(-1.5,0.2)	0.13	1999-2011	1.5*(0.5,2.6)	0.01	2011-2020	-3.5**(-5.1,-1.9)	<0.001	NA	NA	NA	NA	NA	NA
Breast	0.5(-0.3,1.3)	0.18	1999-2020	0.5(-0.3,1.3)	0.18	NA	NA	NA	NA	NA	NA	NA	NA	NA
Stomach	-4.2**(-5,-3.4)	<0.001	1999-2020	-4.2**(-5,-3.4)	<0.001	NA	NA	NA	NA	NA	NA	NA	NA	NA
Liver and Intrahepatic Bile Ducts	-0.8(-2.4,0.9)	0.37	1999-2012	1.2(-0.7,3)	0.20	2012-2020	-3.8*(-7.4,-0.1)	0.04	NA	NA	NA	NA	NA	NA
Uterus	1.1*(0.2,2.1)	0.02	1999-2020	1.1*(0.2,2.1)	0.02	NA	NA	NA	NA	NA	NA	NA	NA	NA
Ovary	-1.1*(-2.2,-0.1)	0.04	1999-2020	-1.1*(-2.2,-0.1)	0.04	NA	NA	NA	NA	NA	NA	NA	NA	NA
Leukemia	-0.6(-1.8,0.7)	0.35	1999-2020	-0.6(-1.8,0.7)	0.35	NA	NA	NA	NA	NA	NA	NA	NA	NA
Kidney and Renal Pelvis	NA	NA	NA	NA	NA	NA	NA	NA	NA	NA	NA	NA	NA	NA
Non-Hodgkin Lymphoma	-2.0**(-2.7,-1.4)	<0.001	1999-2020	-2.0**(-2.7,-1.4)	<0.001	NA	NA	NA	NA	NA	NA	NA	NA	NA
Myeloma	-2.0*(-3.6,-0.4)	0.02	1999-2020	-2.0*(-3.6,-0.4)	0.02	NA	NA	NA	NA	NA	NA	NA	NA	NA
**85+ years**														
Colon and Rectum	-0.8(-2.3,0.8)	0.33	1999-2003	8.5*(0.2,17.4)	0.05	2003-2020	-2.8**(-3.7,-2.0)	<0.001	NA	NA	NA	NA	NA	NA
Pancreas	0.7(-0.8,2.2)	0.39	1999-2010	3.1*(1.0,5.3)	0.01	2010-2020	-2(-4.3,0.4)	0.10	NA	NA	NA	NA	NA	NA

© 2024 Zhu DT et al. *JAMA Network Open*.

Lung and Bronchus	-0.2(-0.8,0.4)	0.49	1999-2020	-0.2(-0.8,0.4)	0.49	NA	NA	NA	NA	NA	NA	NA	NA	NA
Breast	1.0*(0.3,1.7)	0.01	1999-2020	1.0*(0.3,1.7)	0.01	NA	NA	NA	NA	NA	NA	NA	NA	NA
Stomach	-3.4**(-4.4,-2.4)	<0.001	1999-2020	-3.4**(-4.4,-2.4)	<0.001	NA	NA	NA	NA	NA	NA	NA	NA	NA
Liver and Intrahepatic Bile Ducts	-0.5(-1.7,0.7)	0.42	2000-2020	-0.5(-1.7,0.7)	0.42	NA	NA	NA	NA	NA	NA	NA	NA	NA
Ovary	NA	NA	NA	NA	NA	NA	NA	NA	NA	NA	NA	NA	NA	NA
Leukemia	NA	NA	NA	NA	NA	NA	NA	NA	NA	NA	NA	NA	NA	NA
Non-Hodgkin Lymphoma	-2.3(-4.8,0.3)	0.09	2000-2006	-7.3(-14.4,0.4)	0.06	2006-2020	0(-2.2,2.2)	0.96	NA	NA	NA	NA	NA	NA

Segments were chosen by Joinpoint regression.
Statistically significant values in red. ** indicates the p-value is significant after Holm-Bonferroni correction. * indicates the p-value is <0.05, but not significant after Holm-Bonferroni correction.
Abbreviations: APC, Annual percentage change; NA, not applicable

© 2024 Zhu DT et al. *JAMA Network Open*.

eTable 7. Annual Percent Changes in Cancer Death Rates From 1999 to 2020 Among Asian American and Pacific Islander Men by Cancer Type and Census Region

Cause of Death	Average APC from 1999-2020 (95%CI)	p value	Segment 1		p value	Segment 2		p value	Segment 3		p value	Segment 4		p value	Segment 5	
**Men**																
**Northeast**																
Stomach	-3.2**(-3.6,-2.8)	<0.001	1999 - 2011	-3.9**(-4.4,-3.5)	<0.001	2011 - 2020	-2.3**(-3.0,-1.6)	<0.001	NA	NA	NA	NA	NA	NA	NA	NA
Colon and Rectum	-3.4**(-3.6,-3.1)	<0.001	1999 - 2007	-4.3**(-4.8,-3.7)	<0.001	2007 - 2020	-2.8**(-3.1,-2.6)	<0.001	NA	NA	NA	NA	NA	NA	NA	NA
Liver and Intrahepatic Bile Ducts	1.3**(0.9,1.6)	<0.001	1999 - 2014	2.4**(2.1,2.7)	<0.001	2014 - 2020	-1.6**(-2.6,-0.5)	0.01	NA	NA	NA	NA	NA	NA	NA	NA
Pancreas	-0.1(-0.5,0.4)	0.79	1999 - 2001	-2.9(-7.0,1.3)	0.15	2001 - 2007	0.9(0,1.9)	0.05	2007 - 2020	-0.1(-0.3,0.1)	0.49	NA	NA	NA	NA	NA
Lung and Bronchus	-3.4**(-3.6,-3.3)	<0.001	1999 - 2020	-3.4**(-3.6,-3.3)	<0.001	2012 - 2020	-5.1**(-5.5,-4.8)	<0.001	NA	NA	NA	NA	NA	NA	NA	NA
Prostate	-2.8**(-3.1,-2.5)	<0.001	1999 - 2012	-3.6**(-4,-3.3)	<0.001	2012 - 2020	-1.5**(-2.2,-0.8)	<0.001	NA	NA	NA	NA	NA	NA	NA	NA
Esophagus	-0.9**(-1.2,-0.7)	<0.001	1999 - 2005	1.0*(0.1,2.0)	0.04	2005 - 2020	-1.7**(-1.9,-1.5)	<0.001	NA	NA	NA	NA	NA	NA	NA	NA
Kidney and Renal Pelvis	-1.6**(-2.1,-1.2)	<0.001	1999 - 2014	-1.1**(-1.4,-0.7)	<0.001	2014 - 2020	-3.1**(-4.5,-1.7)	<0.001	NA	NA	NA	NA	NA	NA	NA	NA

© 2024 Zhu DT et al. *JAMA Network Open*.

Bladder	-1.2**(-1.9,-0.6)	<0.001	1999-2003	-2**(-3.1,-0.9)	0.003	2003 - 2010	0.8*(0.2,1.4)	0.02	2010 - 2013	-2.2(-5.5,1.3)	0.18	2013 - 2016	-0.2(-3.6,3.3)	0.91	2016 - 2020	-3.9**(-5.0,-2.9)
Brain and other Central Nervous System	0.3(-0.8,1.4)	0.59	1999 - 2013	-0.1(-0.5,0.2)	0.43	2013 - 2016	3.6(-4,11.8)	0.34	2016 - 2020	-0.6(-2.9,1.9)	0.62	NA	NA	NA	NA	NA
Non-Hodgkin Lymphoma	-2.7**(-3.1,-2.3)	<0.001	1999 - 2007	-3.4**(-4.3,-2.4)	<0.001	2007 - 2020	-2.3**(-2.7,-1.8)	<0.001	NA	NA	NA	NA	NA	NA	NA	NA
Leukemia	-1.3**(-1.5,-1.1)	<0.001	1999 - 2011	-0.7**(-1.0,-0.4)	<0.001	2011 - 2020	-2.1**(-2.5,-1.7)	<0.001	NA	NA	NA	NA	NA	NA	NA	NA
Myeloma	-1.0**(-1.2,-0.8)	<0.001	1999 - 2020	-1.0**(-1.2,-0.8)	<0.001	NA	NA	NA	NA	NA	NA	NA	NA	NA	NA	NA
**Midwest**																
Stomach	-2.9**(-3.1,-2.7)	<0.001	1999 - 2020	-2.9**(-3.1,-2.7)	<0.001	NA	NA	NA	NA	NA	NA	NA	NA	NA	NA	NA
Colon and Rectum	-2.5**(-2.9,-2.1)	<0.001	1999 - 2002	-2.2**(-3.4,-1.0)	0.002	2002 - 2005	-4.1**(-6.5,-1.6)	0.004	2005 - 2014	-2.5**(-2.8,-2.3)	<0.001	2014 - 2020	-1.8**(-2.2,-1.4)	<0.001	NA	NA
Liver and Intrahepatic Bile Ducts	1.8**(1.2,2.4)	<0.001	1999 - 2007	1.9**(1.2,2.6)	<0.001	2007 - 2012	3.6**(1.5,5.7)	0.002	2012 - 2020	0.6(-0.1,1.3)	0.07	NA	NA	NA	NA	NA
Pancreas	0.6**(0.5,0.7)	<0.001	1999 - 2020	0.6**(0.5,0.7)	<0.001	NA	NA	NA	NA	NA	NA	NA	NA	NA	NA	NA
Lung and Bronchus	-2.9**(-3.1,-2.6)	<0.001	1999 -	-0.5(-2.6,1.6)	0.59	2001 -	-1.8**(	<0.001	2007 -	-2.7**(	<0.001	2014 -	-4.9**(	<0.001	NA	NA

© 2024 Zhu DT et al. *JAMA Network Open*.

			2001			2007	-2.3,-1.4)		2014	-3.1,-2.4)		2020	-5.2,-4.5)			
Prostate	-2.5**(-2.7,-2.2)	<0.001	1999 - 2013	-3.5**(-3.7,-3.4)	<0.001	2013 - 2020	-0.2(-0.8,0.3)	0.42	NA	NA	NA	NA	NA	NA	NA	NA
Esophagus	-0.2(-0.6,0.2)	0.32	1999 - 2005	0.9(-0.3,2.1)	0.15	2005 - 2020	-0.6**(-0.9,-0.3)	<0.001	NA	NA	NA	NA	NA	NA	NA	NA
Kidney and Renal Pelvis	-0.8(-2.0,0.4)	0.17	1999 - 2001	5.2(-2.3,13.2)	0.16	2001 - 2004	-3.2(-10,4.2)	0.35	2004 - 2013	-0.5(-1.3,0.3)	0.21	2013 - 2020	-1.9**(-2.9,-1)	0.001	NA	NA
Bladder	-0.3*(-0.6,-0.1)	0.02	1999 - 2010	0.3(-0.1,0.7)	0.13	2010 - 2020	-1.1**(-1.5,-0.6)	<0.001	NA	NA	NA	NA	NA	NA	NA	NA
Brain and other Central Nervous System	-0.2(-0.5,0.1)	0.24	1999 - 2003	-2*(-3.6,-0.3)	0.02	2003 - 2020	0.2*(0,0.4)	0.02	NA	NA	NA	NA	NA	NA	NA	NA
Non-Hodgkin Lymphoma	-2.6**(-2.9,-2.2)	<0.001	1999 - 2004	-4**(-5.5,-2.5)	<0.001	2004 - 2020	-2.1**(-2.4,-1.8)	<0.001	NA	NA	NA	NA	NA	NA	NA	NA
Leukemia	-1.3**(-1.5,-1.1)	<0.001	1999 - 2009	-0.7**(-1.0,-0.3)	0.00	2009 - 2020	-1.9**(-2.2,-1.6)	<0.001	NA	NA	NA	NA	NA	NA	NA	NA
Myeloma	-0.9**(-1.4,-0.5)	<0.001	1999 - 2014	-0.6**(-0.9,-0.2)	0.00	2014 - 2020	-1.8*(-3.1,-0.5)	0.01	NA	NA	NA	NA	NA	NA	NA	NA
**South**																
Stomach	-2.7**(-3,-2.4)	<0.001	1999 - 2006	-3.4**(-4.3,-2.6)	<0.001	2006 - 2020	-2.3**(-2.7,-2.0)	<0.001	NA	NA	NA	NA	NA	NA	NA	NA

© 2024 Zhu DT et al. *JAMA Network Open*.

Colon and Rectum	-2**(-2.4,-1.7)	<0.001	1999 - 2001	-0.9(-4.3,2.6)	0.58	2001 - 2011	-2.6**(-2.9,-2.3)	<0.001	2011 - 2020	-1.7**(-2,-1.4)	<0.001	NA	NA	NA	NA	NA
Liver and Intrahepatic Bile Ducts	2.1**(1.8,2.3)	<0.001	1999 - 2014	2.9**(2.8,3.1)	<0.001	2014 - 2020	-0.1(-0.8,0.6)	0.79	NA	NA	NA	NA	NA	NA	NA	NA
Pancreas	0.1(-0.3,0.4)	0.74	1999 - 2001	-1.7(-5.3,2.1)	0.36	2001 - 2020	0.2**(0.1,0.4)	<0.001	NA	NA	NA	NA	NA	NA	NA	NA
Lung and Bronchus	-3.4**(-3.6,-3.2)	<0.001	1999 - 2005	-1.9**(-2.3,-1.5)	<0.001	2005 - 2013	-3.1**(-3.5,-2.8)	<0.001	2013 - 2020	-4.9**(-5.2,-4.6)	<0.001	NA	NA	NA	NA	NA
Prostate	-2.7**(-3.1,-2.3)	<0.001	1999 - 2010	-3.6**(-3.8,-3.4)	<0.001	2010 - 2013	-4.4**(-7.2,-1.5)	<0.001	2013 - 2020	-0.6**(-1,-0.2)	0.004	NA	NA	NA	NA	NA
Esophagus	-0.9**(-1.2,-0.6)	<0.001	1999 - 2007	-0.1(-0.8,0.6)	0.77	2007 - 2020	-1.3**(-1.6,-1)	<0.001	NA	NA	NA	NA	NA	NA	NA	NA
Kidney and Renal Pelvis	-0.7**(-1.1,-0.4)	<0.001	1999 - 2013	-0.4*(-0.7,0)	0.03	2013 - 2020	-1.5**(-2.4,-0.5)	0.01	NA	NA	NA	NA	NA	NA	NA	NA
Bladder	-0.2(-0.4,0.1)	0.28	1999 - 2013	0.4**(0.1,0.7)	0.01	2013 - 2020	-1.3**(-2.0,-0.5)	0.003	NA	NA	NA	NA	NA	NA	NA	NA
Brain and other Central Nervous System	-0.4(-1.0,0.2)	0.19	1999 - 2001	0.2(-4.5,5.1)	0.93	2001 - 2006	-2.9**(-4.4,-1.4)	0.001	2006 - 2012	1.2*(0.1,2.3)	0.04	2012 - 2020	-0.2(-0.7,0.4)	0.50	NA	NA
Non-Hodgkin Lymphoma	-2.6**(-2.9,-2.2)	<0.001	1999 - 2010	-3**(-3.4,-2.5)	<0.001	2010 - 2020	-2.1**(-2.6,-1.6)	<0.001	NA	NA	NA	NA	NA	NA	NA	NA

© 2024 Zhu DT et al. *JAMA Network Open*.

Leukemia	-1.6**(-1.9,-1.4)	<0.001	1999 - 2013	-1.1**(-1.3,-0.9)	<0.001	2013 - 2020	-2.6**(-3.2,-2.1)	<0.001	NA	NA	NA	NA	NA	NA	NA	NA
Myeloma	-1.0**(-1.2,-0.8)	<0.001	1999 - 2020	-1.0**(-1.2,-0.8)	<0.001	NA	NA	NA	NA	NA	NA	NA	NA	NA	NA	NA
**West**																
Stomach	-2.6**(-2.9,-2.3)	<0.001	1999 - 2008	-3.2**(-3.7,-2.7)	<0.001	2008 - 2020	-2.1**(-2.5,-1.8)	<0.001	NA	NA	NA	NA	NA	NA	NA	NA
Colon and Rectum	-2.2**(-2.4,-2)	<0.001	1999 - 2013	-2.6**(-2.7,-2.4)	<0.001	2013 - 2020	-1.5**(-2.1,-1.0)	<0.001	NA	NA	NA	NA	NA	NA	NA	NA
Liver and Intrahepatic Bile Ducts	1.7**(1.3,2.1)	<0.001	1999 - 2013	2.7**(2.4,3.1)	<0.001	2013 - 2020	-0.4(-1.3,0.6)	0.45	NA	NA	NA	NA	NA	NA	NA	NA
Pancreas	0.2*(0.0,0.3)	0.02	1999 - 2020	0.2*(0.0,0.3)	0.02	NA	NA	NA	NA	NA	NA	NA	NA	NA	NA	NA
Lung and Bronchus	-3.8**(-4,-3.6)	<0.001	1999 - 2009	-2.7**(-2.9,-2.5)	<0.001	2009 - 2015	-4.4**(-5.0,-3.8)	<0.001	2015 - 2020	-5.4**(-6.0,-4.8)	<0.001	NA	NA	NA	NA	NA
Prostate	-1.9**(-2.2,-1.6)	<0.001	1999 - 2013	-2.8**(-3.1,-2.5)	<0.001	2013 - 2020	-0.1(-1.0,0.7)	0.77	NA	NA	NA	NA	NA	NA	NA	NA
Esophagus	-1.1**(-1.4,-0.8)	<0.001	1999 - 2012	-0.7**(-1,-0.4)	<0.001	2012 - 2020	-1.7**(-2.4,-1.1)	<0.001	NA	NA	NA	NA	NA	NA	NA	NA
Kidney and Renal Pelvis	-0.8**(-1.0,-0.6)	<0.001	1999 -	-0.8**(	<0.001	NA	NA	NA	NA	NA	NA	NA	NA	NA	NA	NA

© 2024 Zhu DT et al. *JAMA Network Open*.

			2020	-1.0,-0.6)												
Bladder	-0.4(-1.3,0.5)	0.39	1999 - 2005	1.1*(0.2,2)	0.03	2005 - 2008	-2.5(-7.7,2.9)	0.32	2008 - 2012	1.3(-1.4,4.1)	0.32	2012 - 2020	-1.5**(-2.1,-0.9)	<0.001	NA	NA
Brain and other Central Nervous System	-0.2(-0.6,0.2)	0.35	1999 - 2007	-0.9(-1.8,0.1)	0.06	2007 - 2020	0.2(-0.2,0.7)	0.32	NA	NA	NA	NA	NA	NA	NA	NA
Non-Hodgkin Lymphoma	-2.6**(-2.9,-2.2)	<0.001	1999 - 2010	-3.0**(-3.4,-2.5)	<0.001	2010 - 2020	-2.1**(-2.6,-1.6)	<0.001	NA	NA	NA	NA	NA	NA	NA	NA
Leukemia	-1.6**(-1.9,-1.4)	<0.001	1999 - 2013	-1.1**(-1.3,-0.9)	<0.001	2013 - 2020	-2.6**(-3.2,-2.1)	<0.001	NA	NA	NA	NA	NA	NA	NA	NA
Myeloma	-1.0**(-1.2,-0.8)	<0.001	1999 - 2020	-1.0**(-1.2,-0.8)	<0.001	NA	NA	NA	NA	NA	NA	NA	NA	NA	NA	NA

Segments were chosen by Joinpoint regression.
Statistically significant values in red. ** indicates the p-value is significant after Holm-Bonferroni correction. * indicates the p-value is <0.05, but not significant after Holm-Bonferroni correction.
Abbreviations: APC, Annual percentage change; NA, not applicable

© 2024 Zhu DT et al. *JAMA Network Open*.

eTable 8. Annual Percent Changes in Cancer Death Rates From 1999 to 2020 Among Asian American and Pacific Islander Women by Cancer Type and Census Region

**Cause of Death**	**Average APC from 1999-2020 (95%CI)**	**p value**	**Segment 1**		**p value**	**Segment 2**		**p value**	**Segment 3**		**p value**	**Segment 4**		**p value**	**Segment 5**		**p value**
**Women**																	
**Northeast**																	
Colon and Rectum	-3.2**(-3.4,-3.0)	<0.001	1999 - 2009	-3.6**(-3.9,-3.2)	<0.001	2009 - 2020	-2.8**(-3.1,-2.5)	<0.001	NA	NA	NA	NA	NA	NA	NA	NA	NA
Pancreas	0.1(0.0,0.1)	0.24	1999 - 2020	0.1(0.0,0.1)	0.24	NA	NA	NA	NA	NA	NA	NA	NA	NA	NA	NA	NA
Lung and Bronchus	-2.0**(-2.3,-1.7)	<0.001	1999 - 2002	0.9(-0.5,2.4)	0.18	2002 - 2010	-1.0**(-1.4,-0.7)	<0.001	2010 - 2015	-2.5**(-3.4,-1.6)	<0.001	2015 - 2020	-4.8**(-5.4,-4.2)	<0.001	NA	NA	NA
Breast	-2.1**(-2.3,-2)	<0.001	1999 - 2014	-2.3**(-2.5,-2.2)	<0.001	2014 - 2020	-1.6**(-2.1,-1.1)	<0.001	NA	NA	NA	NA	NA	NA	NA	NA	NA
Stomach	-3.0**(-4.2,-1.8)	<0.001	1999 - 2003	-2.2(-4.7,0.5)	0.10	2003 - 2006	-6.4(-13.9,1.8)	0.11	2006 - 2020	-2.5**(-2.9,-2.1)	<0.001	NA	NA	NA	NA	NA	NA
Liver and Intrahepatic Bile Ducts	1.5**(0.9,2.1)	<0.001	1999 - 2013	2.1**(1.6,2.6)	<0.001	2013 - 2020	0.3(-1.2,1.8)	0.70	NA	NA	NA	NA	NA	NA	NA	NA	NA
Cervix	-2.4**(-3.5,-1.2)	<0.001	1999 - 2001	-7.5(-18.7,5.3)	0.22	2001 - 2020	-1.8**(-2.2,-1.4)	<0.001	NA	NA	NA	NA	NA	NA	NA	NA	NA
Uterus	1.2**(1.0,1.4)	<0.001	1999 -	1.2**(1.0,1.4)	<0.001	NA	NA	NA	NA	NA	NA	NA	NA	NA	NA	NA	NA

© 2024 Zhu DT et al. *JAMA Network Open*.

			2020														
Ovary	-2.1**(-2.3,-1.8)	<0.001	1999 - 2006	-0.9*(-1.6,-0.1)	0.03	2006 - 2020	-2.7**(-2.9,-2.4)	<0.001	NA	NA	NA	NA	NA	NA	NA	NA	NA
Leukemia	-1.6**(-1.9,-1.3)	<0.001	1999 - 2013	-1.1**(-1.4,-0.8)	<0.001	2013 - 2020	-2.6**(-3.5,-1.8)	<0.001	NA	NA	NA	NA	NA	NA	NA	NA	NA
Esophagus	-1.7**(-2.1,-1.4)	<0.001	1999 - 2020	-1.7**(-2.1,-1.4)	<0.001	NA	NA	NA	NA	NA	NA	NA	NA	NA	NA	NA	NA
Kidney and Renal Pelvis	-1.9**(-2.5,-1.3)	<0.001	1999 - 2004	-0.1(-2.5,2.4)	0.93	2004 - 2020	-2.4**(-2.8,-2.0)	<0.001	NA	NA	NA	NA	NA	NA	NA	NA	NA
Bladder	-1.4**(-1.9,-0.8)	<0.001	1999 - 2016	-0.6**(-0.9,-0.3)	0.001	2016 - 2020	-4.7**(-7.3,-2.1)	0.002	NA	NA	NA	NA	NA	NA	NA	NA	NA
Brain and other Central Nervous System	-0.1(-0.9,0.7)	0.76	1999 - 2007	-1.3*(-2.4,-0.3)	0.02	2007 - 2016	1.5*(0.4,2.6)	0.01	2016 - 2020	-1.4(-4.4,1.8)	0.36	NA	NA	NA	NA	NA	NA
Non-Hodgkin Lymphoma	-3.5**(-4.0,-3.0)	<0.001	1999 - 2002	-5.5*(-9.1,-1.7)	0.01	2002 - 2020	-3.2**(-3.4,-2.9)	<0.001	NA	NA	NA	NA	NA	NA	NA	NA	NA
Myeloma	-2.2**(-3.0,-1.3)	<0.001	1999 - 2008	-2.4**(-3.3,-1.4)	<0.001	2008 - 2016	0.1(-1.4,1.5)	0.93	2016 - 2020	-6**(-9.2,-2.7)	0.002	NA	NA	NA	NA	NA	NA
**Midwest**																	
Colon and Rectum	-2.5**(-2.7,-2.3)	<0.001	1999 - 2009	-3**(-3.3,-2.7)	<0.001	2009 - 2020	-2.1**(-2.4,-1.8)	<0.001	NA	NA	NA	NA	NA	NA	NA	NA	NA

© 2024 Zhu DT et al. *JAMA Network Open*.

Pancreas	0.4**(0.3,0.6)	<0.001	1999 - 2020	0.4**(0.3,0.6)	<0.001	NA	NA	NA	NA	NA	NA	NA	NA	NA	NA	NA	NA
Lung and Bronchus	-1.3**(-1.5,-1.1)	<0.001	1999 - 2002	1.6**(0.6,2.6)	0.004	2002 - 2008	-0.2(-0.6,0.3)	0.37	2008 - 2014	-1.4**(-1.8,-0.9)	<0.001	2014 - 2020	-3.7**(-4.0,-3.4)	<0.001	NA	NA	NA
Breast	-1.7**(-1.9,-1.6)	<0.001	1999 - 2009	-1.9**(-2.2,-1.7)	<0.001	2009 - 2020	-1.5**(-1.7,-1.3)	<0.001	NA	NA	NA	NA	NA	NA	NA	NA	NA
Stomach	-2.4**(-2.7,-2.2)	<0.001	1999 - 2020	-2.4**(-2.7,-2.2)	<0.001	NA	NA	NA	NA	NA	NA	NA	NA	NA	NA	NA	NA
Liver and Intrahepatic Bile Ducts	2.1**(1.8,2.4)	<0.001	1999 - 2020	2.1**(1.8,2.4)	<0.001	NA	NA	NA	NA	NA	NA	NA	NA	NA	NA	NA	NA
Cervix	-1.1**(-1.5,-0.8)	<0.001	1999 - 2005	-2.2**(-3.4,-1.0)	0.002	2005 - 2020	-0.7**(-1,-0.4)	<0.001	NA	NA	NA	NA	NA	NA	NA	NA	NA
Uterus	1.0*(0.1,1.9)	0.03	1999 - 2013	0.7**(0.4,1.0)	<0.001	2013 - 2016	3.4(-2.8,9.9)	0.27	2016 - 2020	0.2(-1.7,2.2)	0.82	NA	NA	NA	NA	NA	NA
Ovary	-1.9**(-2.5,-1.4)	<0.001	1999 - 2002	1.7(-1.2,4.7)	0.23	2002 - 2016	-2.2**(-2.5,-1.9)	<0.001	2016 - 2020	-3.7**(-5.5,-1.9)	<0.001	NA	NA	NA	NA	NA	NA
Leukemia	-1.7**(-2.0,-1.3)	<0.001	1999 - 2004	-1.9**(-3.0,-0.9)	0.001	2004 - 2011	-0.9*(-1.6,-0.1)	0.04	2011 - 2020	-2.2**(-2.6,-1.7)	<0.001	NA	NA	NA	NA	NA	NA
Esophagus	-0.7**(-1.0,-0.5)	<0.001	1999 - 2020	-0.7**(-1.0,-0.5)	<0.001	NA	NA	NA	NA	NA	NA	NA	NA	NA	NA	NA	NA

© 2024 Zhu DT et al. *JAMA Network Open*.

Kidney and Renal Pelvis	-1.6**(-2.0,-1.1)	<0.001	1999 - 2012	-1.0**(-1.5,-0.4)	0.001	2012 - 2020	-2.5**(-3.6,-1.4)	<0.001	NA	NA	NA	NA	NA	NA	NA	NA	NA
Bladder	-0.6**(-1.0,-0.2)	0.001	1999 - 2013	-0.1(-0.4,0.3)	0.61	2013 - 2020	-1.6**(-2.6,-0.7)	0.002	NA	NA	NA	NA	NA	NA	NA	NA	NA
Brain and other Central Nervous System	-0.2(-0.4,0.0)	0.10	1999 - 2020	-0.2(-0.4,0.0)	0.10	NA	NA	NA	NA	NA	NA	NA	NA	NA	NA	NA	NA
Non-Hodgkin Lymphoma	-3.0**(-3.6,-2.4)	<0.001	1999 - 2006	-4.2**(-4.8,-3.6)	<0.001	2006 - 2010	-1.9(-4.1,0.5)	0.11	2010 - 2018	-3.2**(-3.8,-2.6)	<0.001	2018 - 2020	0.2(-4.4,5.0)	0.93	NA	NA	NA
Myeloma	-1.3**(-2.1,-0.5)	0.002	1999 - 2001	0.6(-4.4,5.9)	0.80	2001 - 2009	-2.4**(-3.1,-1.8)	<0.001	2009 - 2012	1.3(-3.7,6.6)	0.59	2012 - 2020	-1.6**(-2.1,-1,0)	<0.001	NA	NA	NA
**South**																	
Colon and Rectum	-2.3**(-2.5,-2.0)	<0.001	1999 - 2012	-2.6**(-2.8,-2.4)	<0.001	2012 - 2020	-1.7**(-2.2,-1.3)	<0.001	NA	NA	NA	NA	NA	NA	NA	NA	NA
Pancreas	0.2**(0.2,0.3)	<0.001	1999 - 2020	0.2**(0.2,0.3)	<0.001	NA	NA	NA	NA	NA	NA	NA	NA	NA	NA	NA	NA
Lung and Bronchus	-1.8**(-2.0,-1.5)	<0.001	1999 - 2001	1.8(-0.2,3.8)	0.07	2001 - 2005	-0.1(-1.1,0.9)	0.77	2005 - 2013	-1.7**(-2.0,-1.4)	<0.001	2013 - 2020	-3.8**(-4.0,-3.5)	<0.001	NA	NA	NA
Breast	-1.3**(-1.6,-1.0)	<0.001	1999 - 2002	-0.5(-2.0,1.1)	0.55	2002 - 2008	-2.1**(-2.8,-1.4)	<0.001	2008 - 2020	-1.2**(-1.4,-1)	<0.001	NA	NA	NA	NA	NA	NA
Stomach	-2.2**(-2.6,-1.8)	<0.001	1999 -	-3.2**(	<0.001	2005 -	-1.8**(	<0.001	NA	NA	NA	NA	NA	NA	NA	NA	NA

© 2024 Zhu DT et al. *JAMA Network Open*.

			2005	-4.4,-2.0)		2020	-2.1,-1.5)										
Liver and Intrahepatic Bile Ducts	1.5**(1.1,1.8)	<0.001	1999 - 2006	0.6(0.0,1.3)	0.06	2006 - 2015	2.8**(2.3,3.4)	<0.001	2015 - 2020	0.1(-1.0,1.2)	0.84	NA	NA	NA	NA	NA	NA
Cervix	-1.1**(-1.5,-0.7)	<0.001	1999 - 2004	-3.1**(-4.5,-1.7)	<0.001	2004 - 2020	-0.5**(-0.7,-0.2)	0.002	NA	NA	NA	NA	NA	NA	NA	NA	NA
Uterus	1.0**(0.5,1.6)	<0.001	1999 - 2003	-1.1(-2.6,0.4)	0.14	2003 - 2009	0.6(-0.5,1.7)	0.23	2009 - 2016	2.6**(1.7,3.4)	<0.001	2016 - 2020	1.1(-0.4,2.7)	0.14	NA	NA	NA
Ovary	-1.8**(-2.1,-1.6)	<0.001	1999 - 2005	-0.1(-1.0,0.7)	0.75	2005 - 2020	-2.5**(-2.7,-2.3)	<0.001	NA	NA	NA	NA	NA	NA	NA	NA	NA
Leukemia	-1.4**(-1.6,-1.3)	<0.001	1999 - 2020	-1.4**(-1.6,-1.3)	<0.001	NA	NA	NA	NA	NA	NA	NA	NA	NA	NA	NA	NA
Esophagus	-1.6**(-2.0,-1.2)	<0.001	1999 - 2007	-2.3**(-3.1,-1.4)	<0.001	2007 - 2020	-1.1**(-1.6,-0.7)	<0.001	NA	NA	NA	NA	NA	NA	NA	NA	NA
Kidney and Renal Pelvis	-0.8*(-1.6,0.0)	0.049	1999 - 2001	4.3(-4.5,13.9)	0.33	2001 - 2020	-1.3**(-1.6,-1.1)	<0.001	NA	NA	NA	NA	NA	NA	NA	NA	NA
Bladder	-0.5**(-0.7,-0.3)	<0.001	1999 - 2020	-0.5**(-0.7,-0.3)	<0.001	NA	NA	NA	NA	NA	NA	NA	NA	NA	NA	NA	NA
Brain and other Central Nervous System	-0.4(-0.9,0.2)	0.20	1999 - 2007	-1.3**(-2.0,-0.5)	0.003	2007 - 2015	0.8(-0.1,1.8)	0.08	2015 - 2020	-0.8(-2.3,0.8)	0.31	NA	NA	NA	NA	NA	NA

© 2024 Zhu DT et al. *JAMA Network Open*.

Non-Hodgkin Lymphoma	-3.1**(-3.2,-2.9)	<0.001	1999 - 2020	-3.1**(-3.2,-2.9)	<0.001	NA	NA	NA	NA	NA	NA	NA	NA	NA	NA	NA	NA
Myeloma	-1.5**(-2.2,-0.9)	<0.001	1999 - 2009	-2.3**(-2.8,-1.8)	<0.001	2009 - 2013	1.1(-2.2,4.5)	0.50	2013 - 2020	-2.0**(-2.8,-1.1)	<0.001	NA	NA	NA	NA	NA	NA
**West**																	
Colon and Rectum	-2.2**(-2.4,-2.1)	<0.001	1999 - 2020	-2.2**(-2.4,-2.1)	<0.001	NA	NA	NA	NA	NA	NA	NA	NA	NA	NA	NA	NA
Pancreas	0.1(-0.1,0.3)	0.35	1999 - 2005	0.9**(0.3,1.6)	0.005	2005 - 2020	-0.2**(-0.4,-0.1)	0.003	NA	NA	NA	NA	NA	NA	NA	NA	NA
Lung and Bronchus	-2.8**(-3.3,-2.3)	<0.001	1999 - 2003	-0.6(-1.5,0.3)	0.14	2003 - 2009	-2.1**(-2.7,-1.5)	<0.001	2009 - 2015	-3.0**(-3.6,-2.4)	<0.001	2015 - 2018	-5.8**(-8.3,-3.1)	0.001	2018 - 2020	-4.1**(-6.7,-1.5)	0.01
Breast	-1.5**(-1.6,-1.3)	<0.001	1999 - 2008	-1.9**(-2.2,-1.6)	<0.001	2008 - 2020	-1.2**(-1.4,-1.0)	<0.001	NA	NA	NA	NA	NA	NA	NA	NA	NA
Stomach	-1.7**(-2.2,-1.3)	<0.001	1999 - 2013	-2.2**(-2.6,-1.8)	<0.001	2013 - 2020	-0.7(-1.9,0.5)	0.22	NA	NA	NA	NA	NA	NA	NA	NA	NA
Liver and Intrahepatic Bile Ducts	1.9**(1.1,2.7)	<0.001	1999 - 2002	5.2**(1.9,8.6)	0.005	2002 - 2009	0.7(-0.4,1.8)	0.18	2009 - 2013	3.6*(0.3,6.9)	0.03	2013 - 2020	0.7(-0.1,1.6)	0.10	NA	NA	NA
Cervix	-1.3**(-2.1,-0.5)	0.001	1999 - 2003	-4.7*(-8.6,-0.6)	0.03	2003 - 2020	-0.5*(-1.0,-0.1)	0.03	NA	NA	NA	NA	NA	NA	NA	NA	NA
Uterus	1.3**(0.9,1.7)	<0.001	1999 -	-0.1(-1.2,1.0)	0.83	2006 -	2.0**(1.6,2.4)	<0.001	NA	NA	NA	NA	NA	NA	NA	NA	NA

© 2024 Zhu DT et al. *JAMA Network Open*.

			2006			2020											
Ovary	-1.9**(-2.4,-1.4)	<0.001	1999 - 2004	0.6(-0.2,1.3)	0.12	2004 - 2007	-3.7*(-6.8,-0.5)	0.03	2007 - 2016	-1.7**(-2.1,-1.4)	<0.001	2016 - 2020	-3.9**(-4.9,-2.9)	<0.001	NA	NA	NA
Leukemia	-1.5**(-1.9,-1.2)	<0.001	1999 - 2010	-1.0**(-1.4,-0.5)	0.001	2010 - 2020	-2.1**(-2.7,-1.6)	<0.001	NA	NA	NA	NA	NA	NA	NA	NA	NA
Esophagus	-1.9**(-2.2,-1.6)	<0.001	1999 - 2020	-1.9**(-2.2,-1.6)	<0.001	NA	NA	NA	NA	NA	NA	NA	NA	NA	NA	NA	NA
Kidney and Renal Pelvis	-1.0**(-1.4,-0.6)	<0.001	1999 - 2005	0.1(-1.3,1.5)	0.88	2005 - 2020	-1.4**(-1.8,-1.1)	<0.001	NA	NA	NA	NA	NA	NA	NA	NA	NA
Bladder	-0.8**(-1.1,-0.5)	<0.001	1999 - 2020	-0.8**(-1.1,-0.5)	<0.001	NA	NA	NA	NA	NA	NA	NA	NA	NA	NA	NA	NA
Brain and other Central Nervous System	-0.4(-1.2,0.3)	0.26	1999 - 2009	-1.2**(-1.8,-0.6)	<0.001	2009 - 2013	1.6(-2.2,5.6)	0.38	2013 - 2020	-0.5(-1.5,0.5)	0.31	NA	NA	NA	NA	NA	NA
Non-Hodgkin Lymphoma	-3.0**(-3.2,-2.8)	<0.001	1999 - 2020	-3.0**(-3.2,-2.8)	<0.001	NA	NA	NA	NA	NA	NA	NA	NA	NA	NA	NA	NA
Myeloma	-1.5**(-1.8,-1.1)	<0.001	1999 - 2020	-1.5**(-1.8,-1.1)	<0.001	NA	NA	NA	NA	NA	NA	NA	NA	NA	NA	NA	NA

Segments were chosen by Joinpoint regression.
Statistically significant values in red. ** indicates the p-value is significant after Holm-Bonferroni correction. * indicates the p-value is <0.05, but not significant after Holm-Bonferroni correction.
Abbreviations: APC, Annual percentage change; NA, not applicable

© 2024 Zhu DT et al. *JAMA Network Open*.

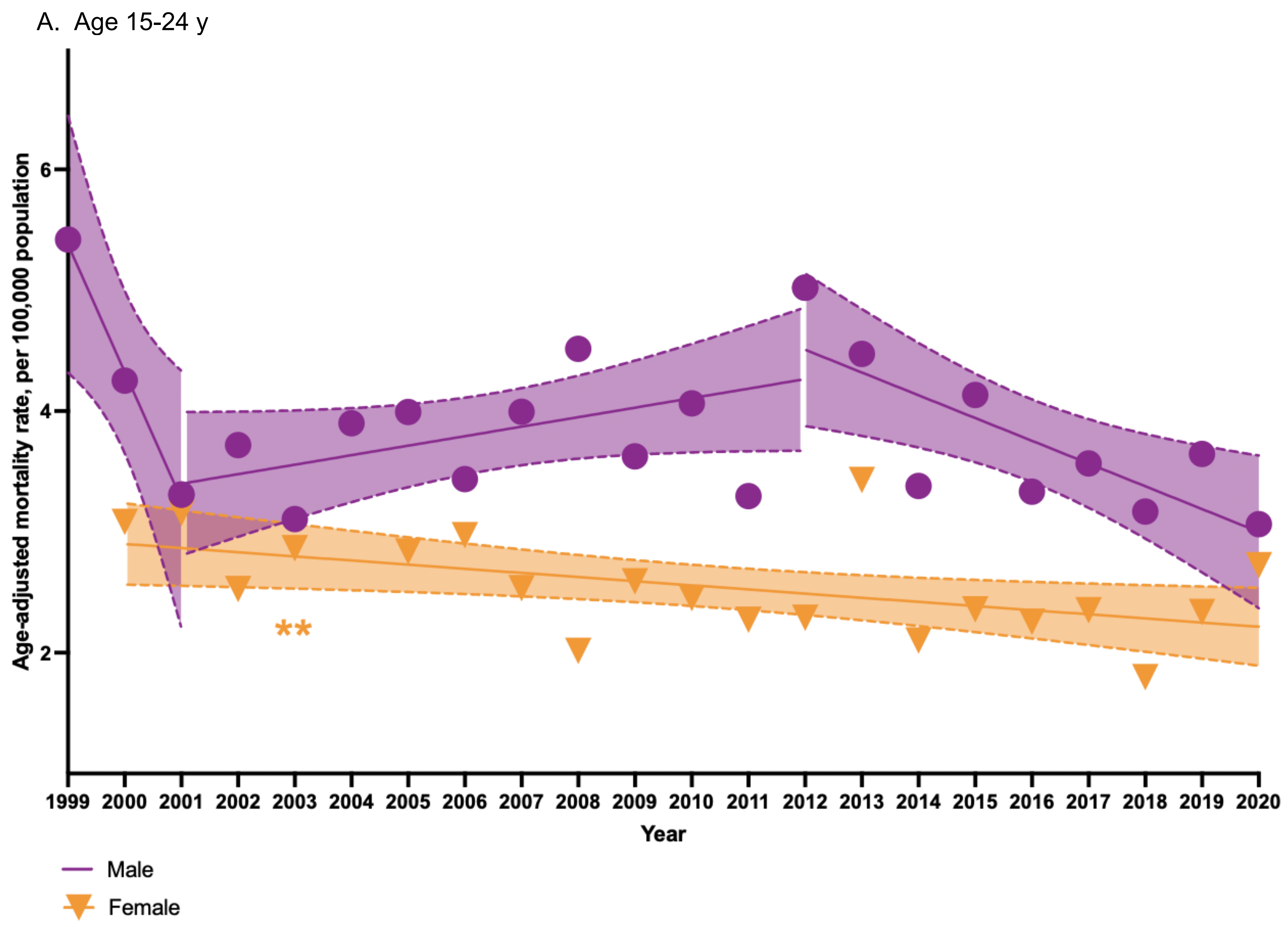

© 2024 Zhu DT et al. *JAMA Network Open*.

B. Age 25-34 y

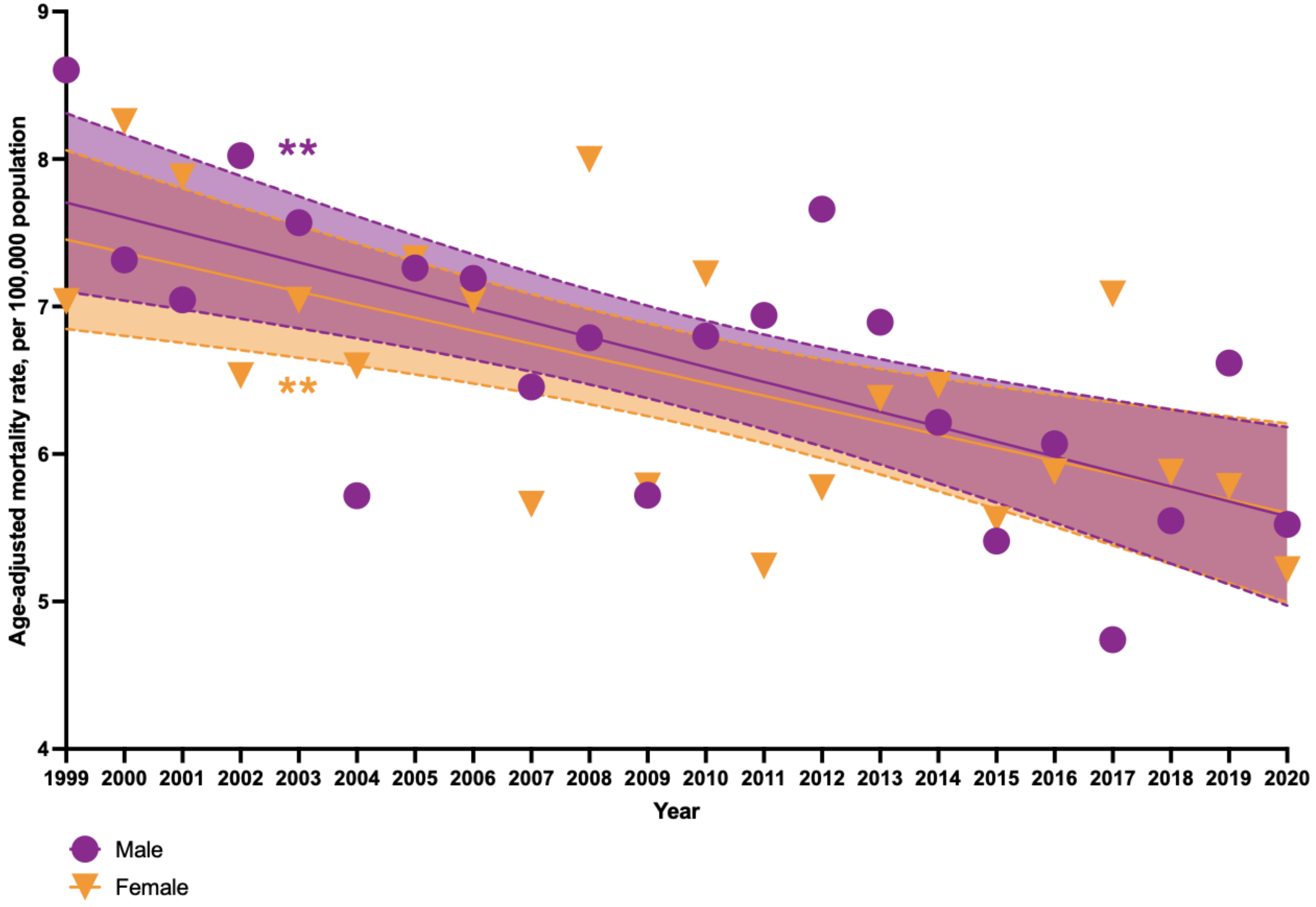

© 2024 Zhu DT et al. *JAMA Network Open*.

C. Age 35-44 y

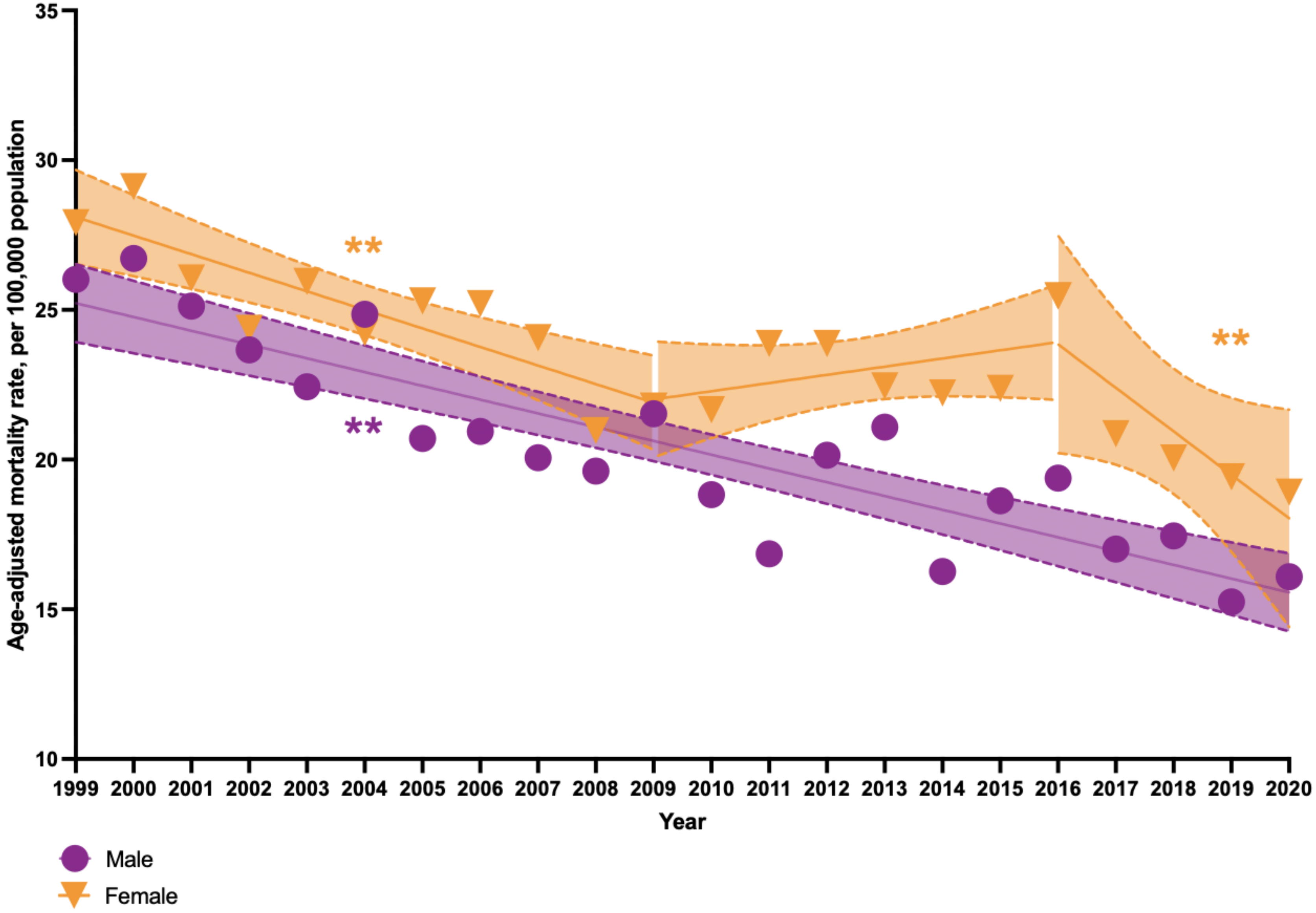

© 2024 Zhu DT et al. *JAMA Network Open*.

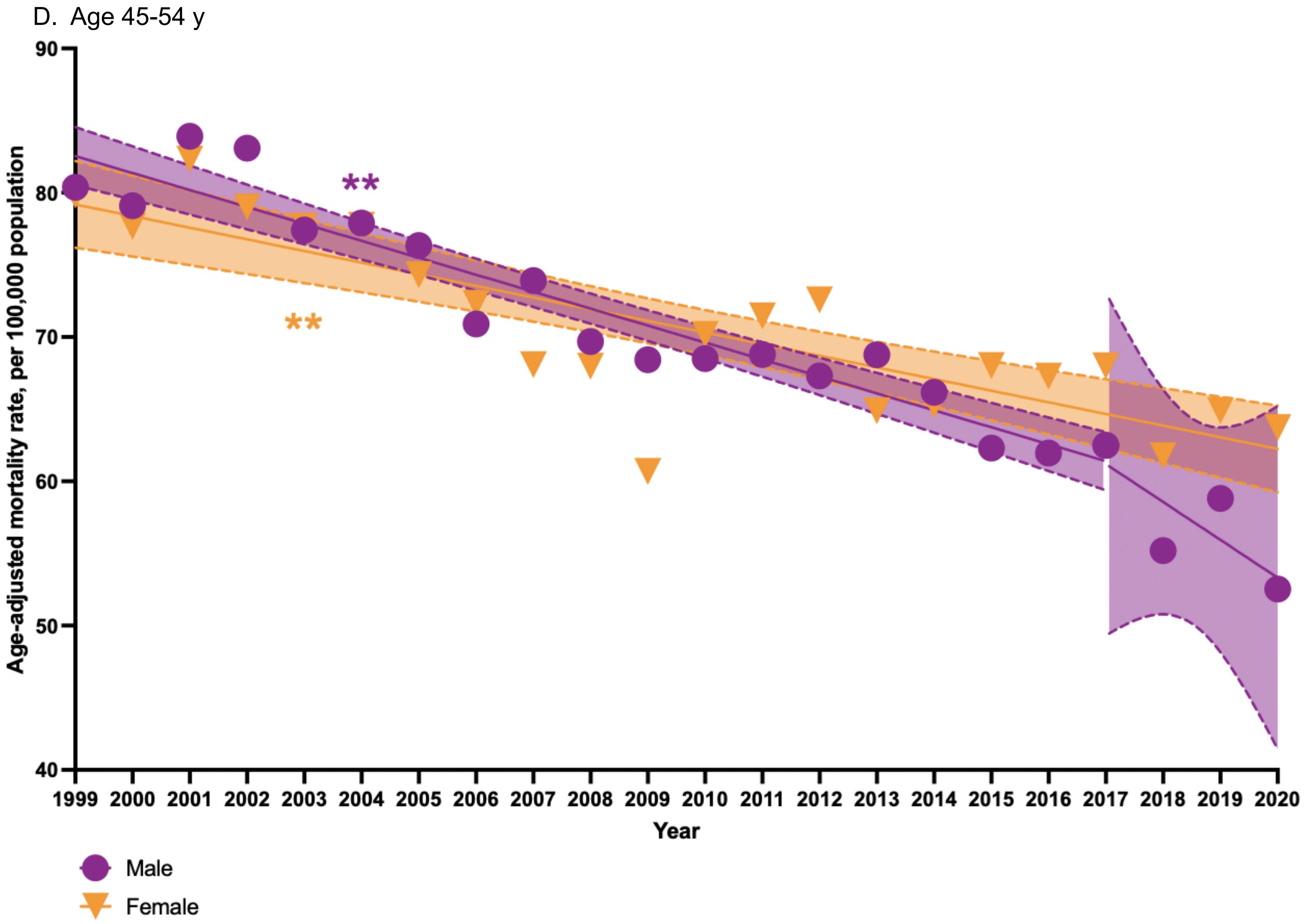

© 2024 Zhu DT et al. *JAMA Network Open*.

E. Age 55-64 y

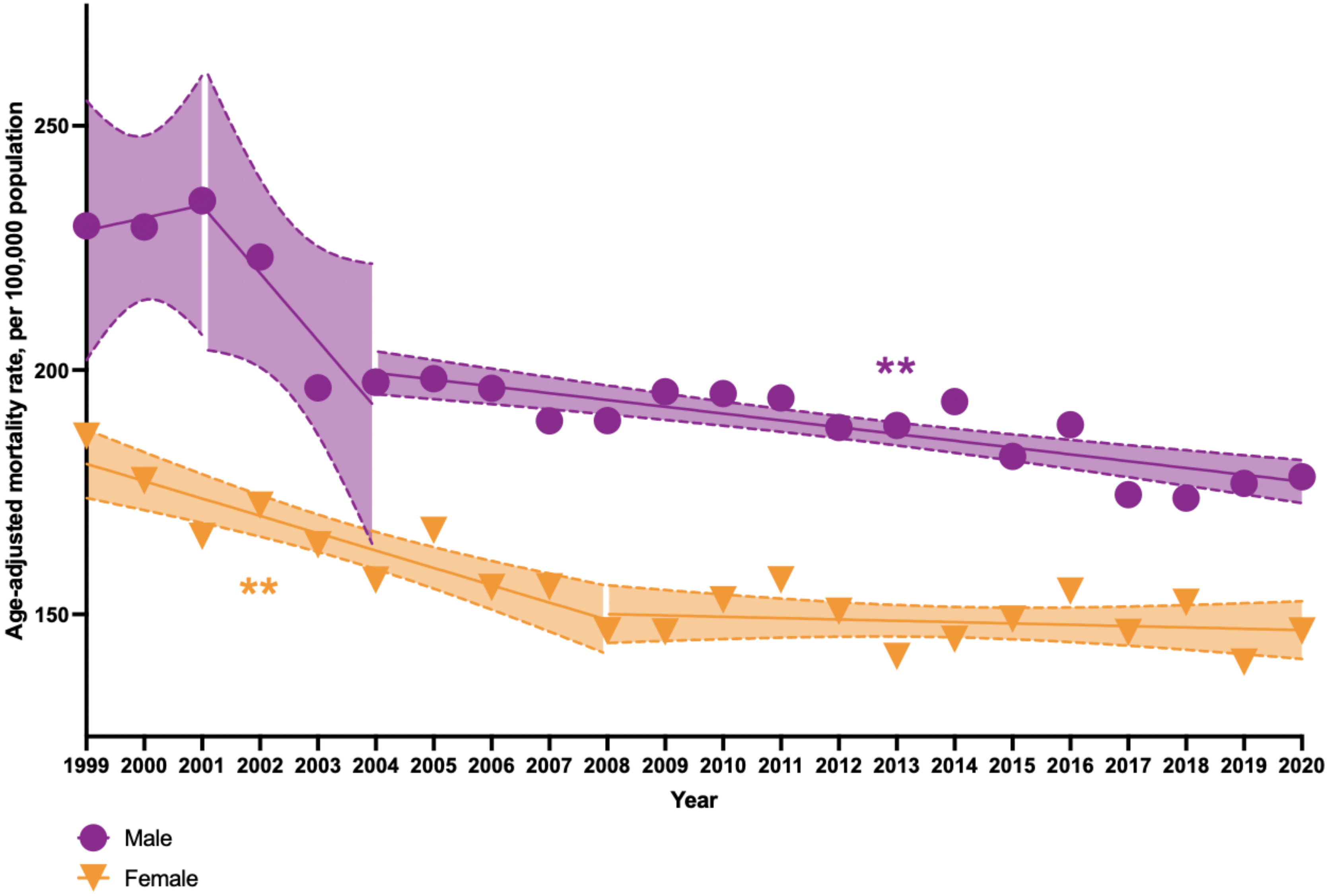

© 2024 Zhu DT et al. *JAMA Network Open*.

F. Age 65-74 y

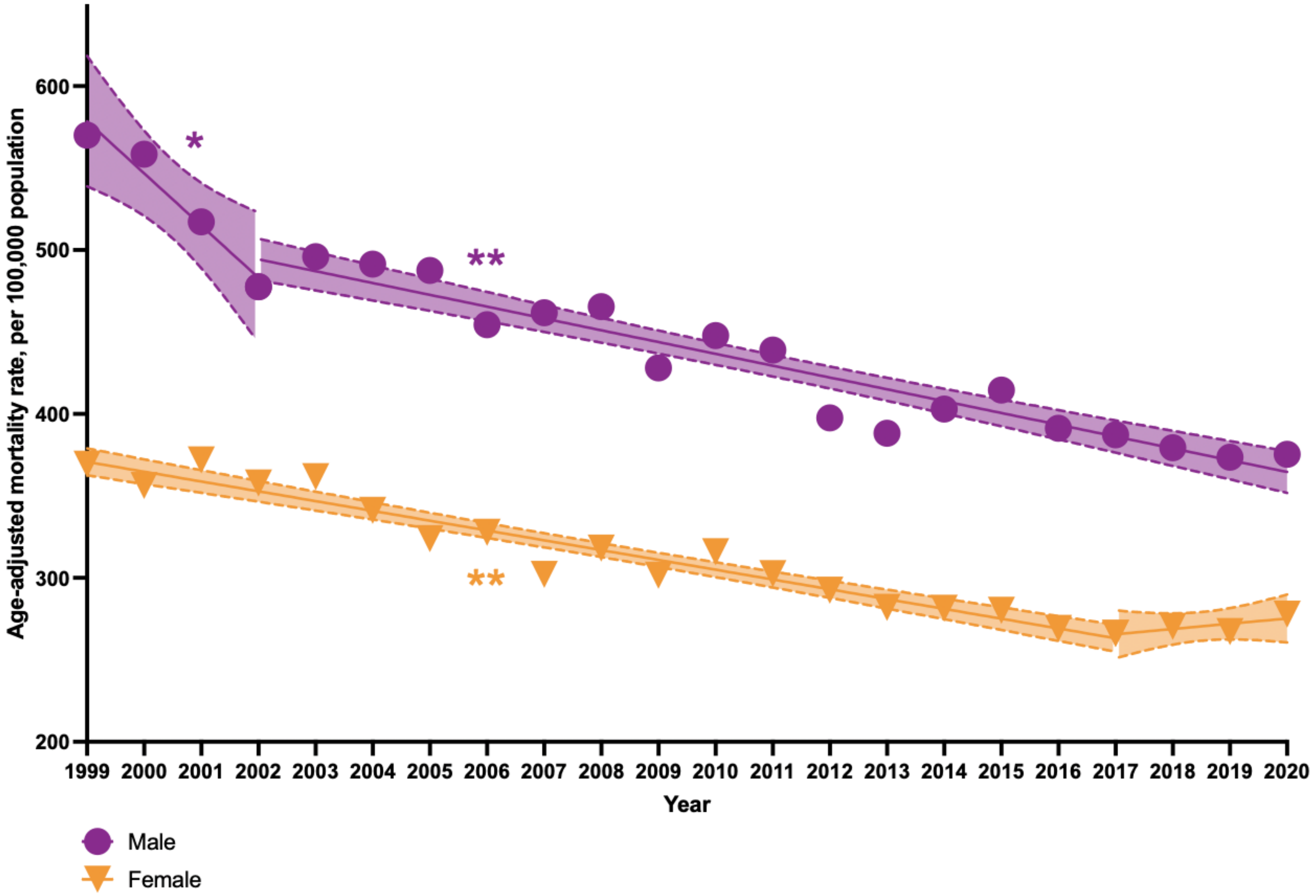

© 2024 Zhu DT et al. *JAMA Network Open*.

G. Age 75-84 y

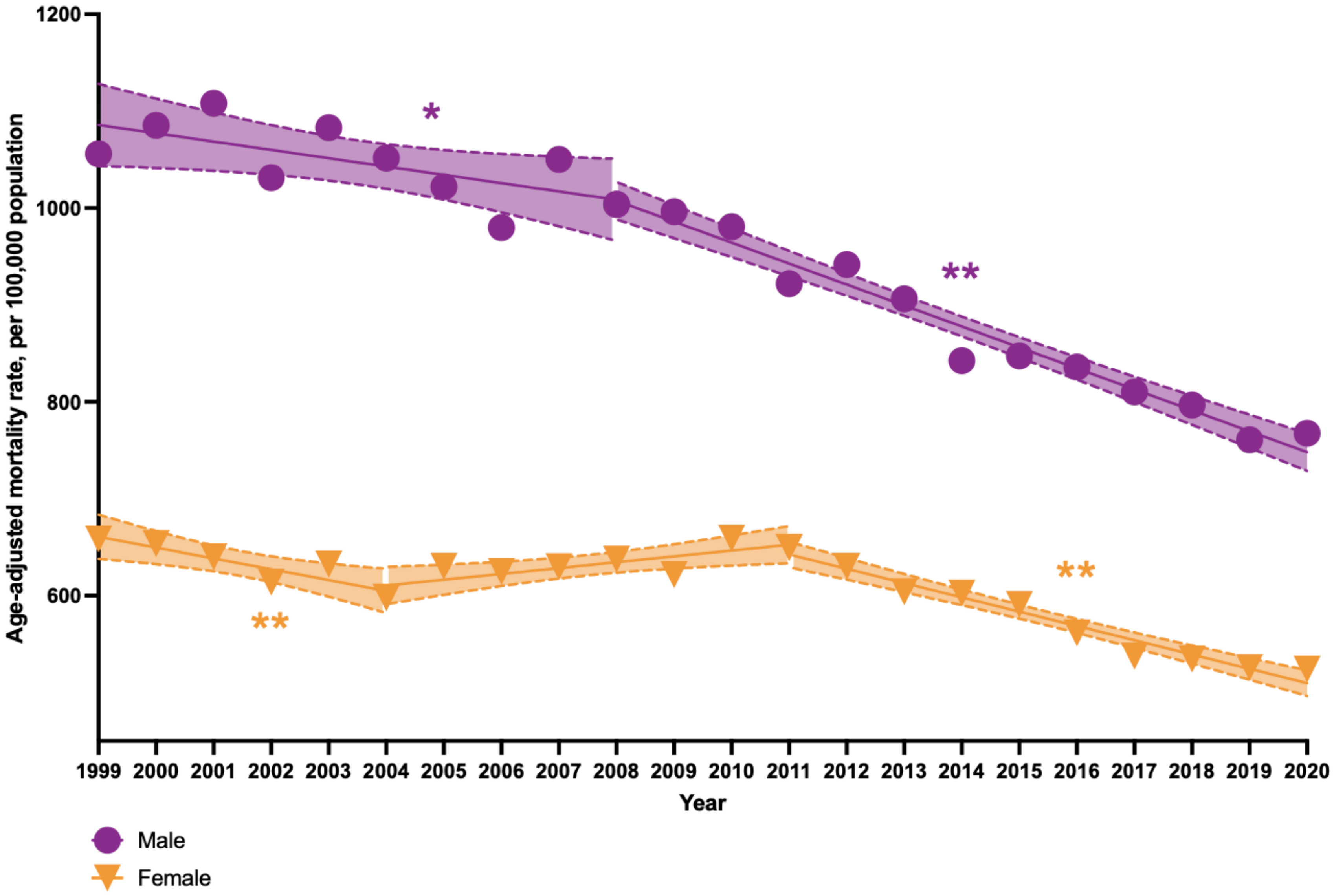

© 2024 Zhu DT et al. *JAMA Network Open*.

H. Age ≥85 y

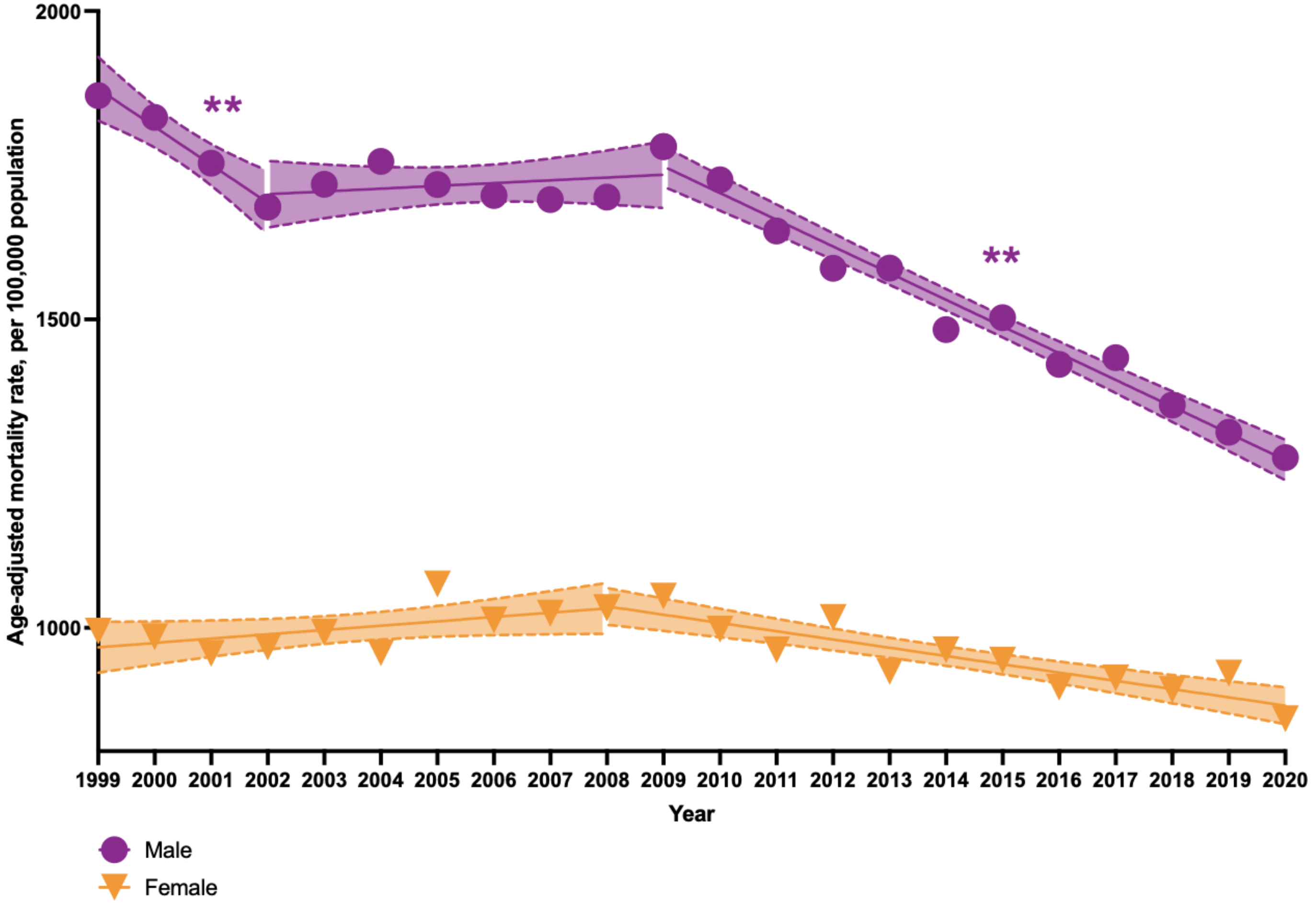

eFigure 1. Trends in Asian American and Pacific Islander Age-Adjusted Cancer Death Rates From 1999 to 2020 by Sex and Age Group. Observed rates are presented per 100,000 population and represented by markers. Modeled trends are represented by “---”. “ ** ” indicates the AAPC p-value is significant after Holm-Bonferroni correction. “ * ” indicates the

© 2024 Zhu DT et al. *JAMA Network Open*.

p-value is <0.05, but not significant after Holm-Bonferroni correction. Confidence bands represent 95% confidence intervals.

A. Northeast

© 2024 Zhu DT et al. *JAMA Network Open*.

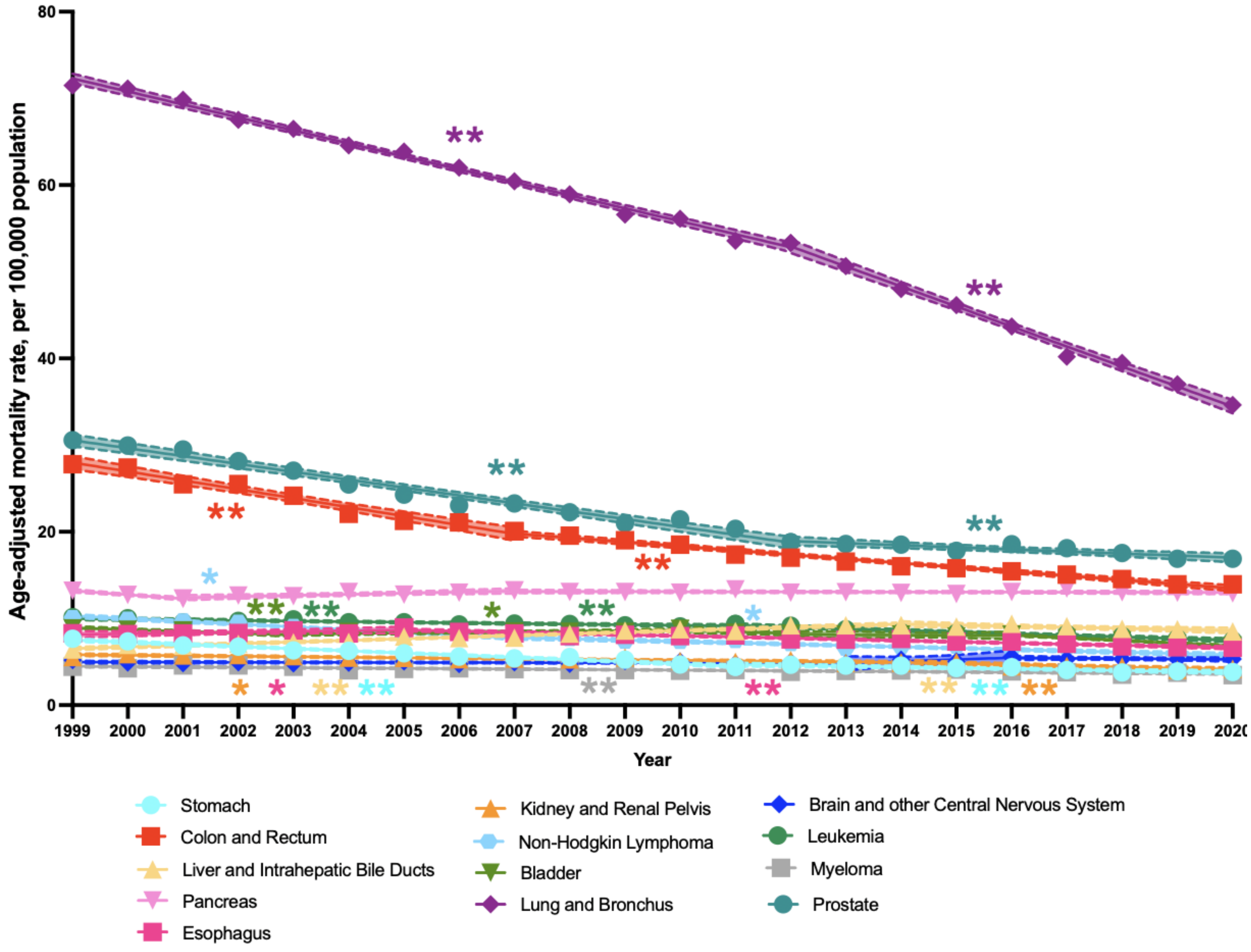

B. Midwest

© 2024 Zhu DT et al. *JAMA Network Open*.

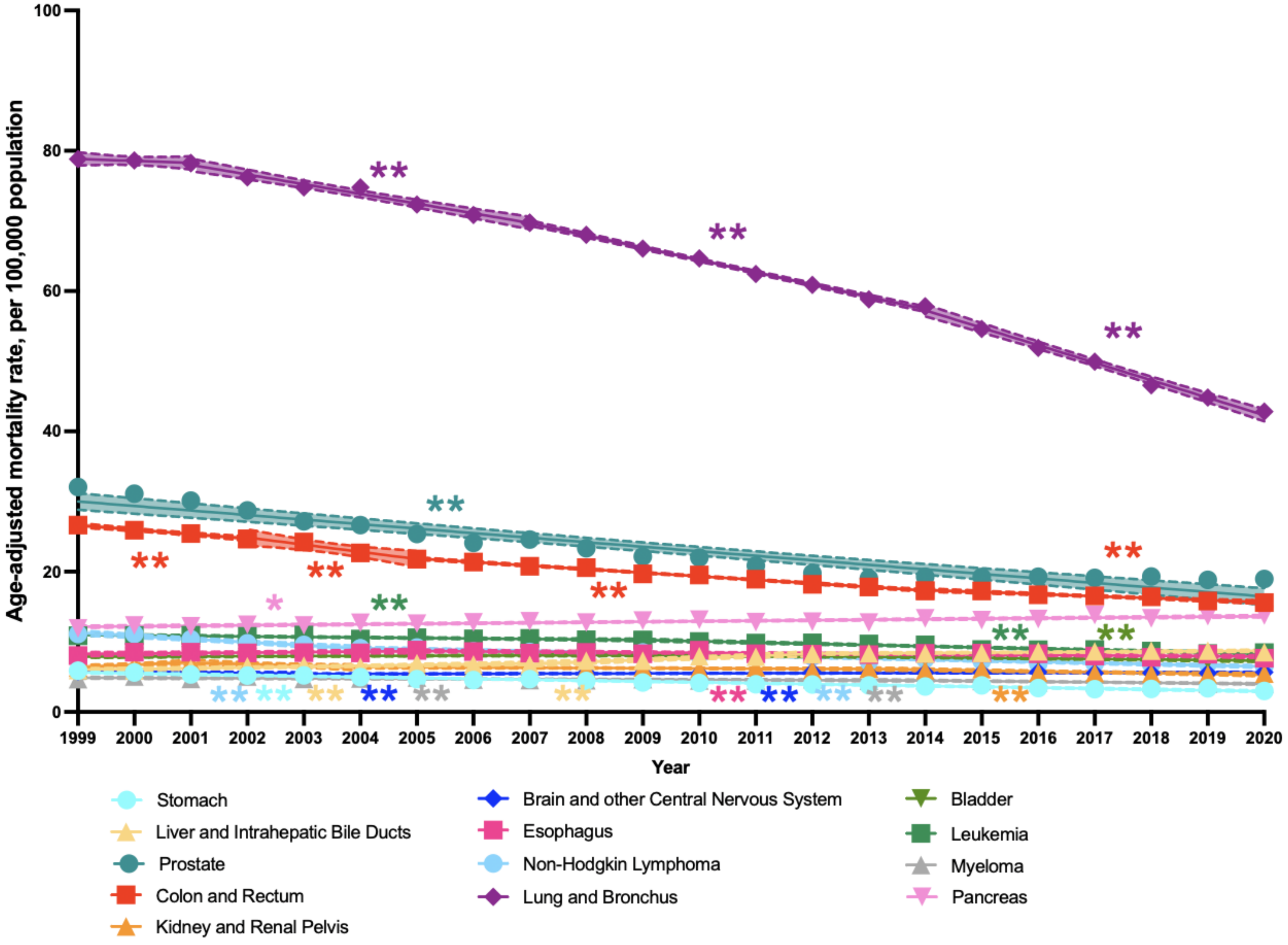

C. South

© 2024 Zhu DT et al. *JAMA Network Open*.

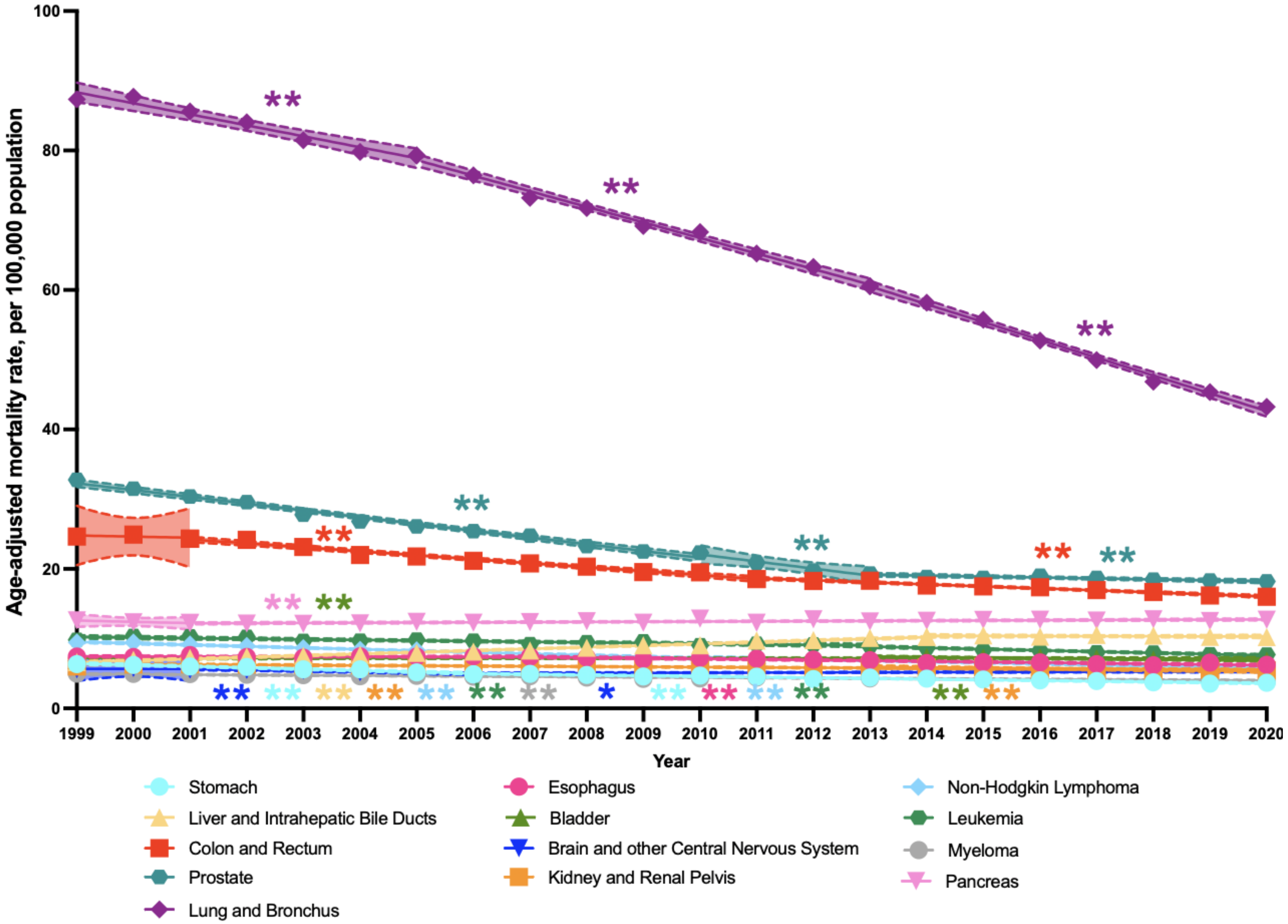

D. West

© 2024 Zhu DT et al. *JAMA Network Open*.

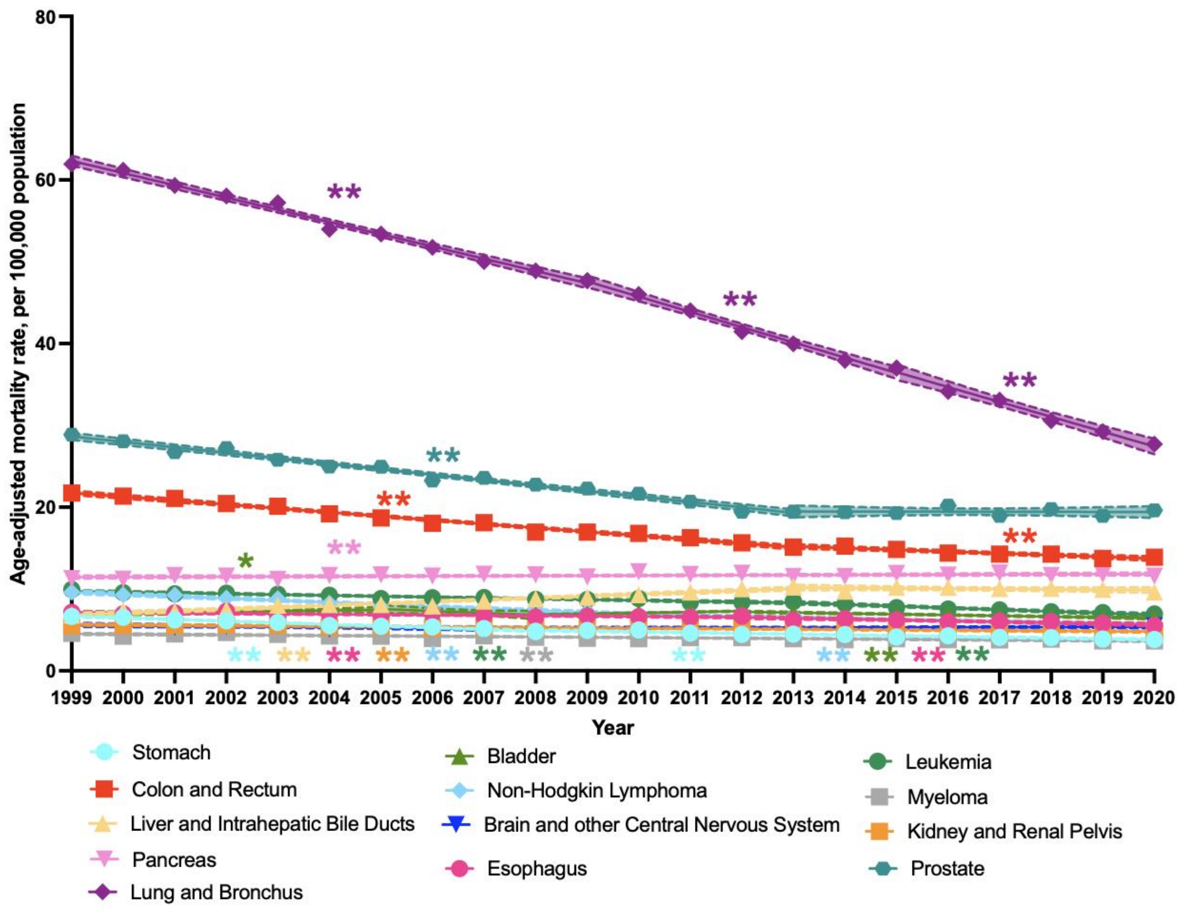

eFigure 2. Trends in Age-Adjusted Death Rates From 1999 to 2020 Among Asian American and Pacific Islander Men by Cancer Type and US Census Region. Observed rates are presented per 100,000 population and represented by markers. Modeled trends are represented by "---". " ** " indicates the AAPC p-value is significant after Holm-Bonferroni correction. " * " indicates the p-value is <0.05, but not significant after Holm-Bonferroni correction. Confidence bands represent 95% confidence intervals.

© 2024 Zhu DT et al. *JAMA Network Open*.

A. Northeast

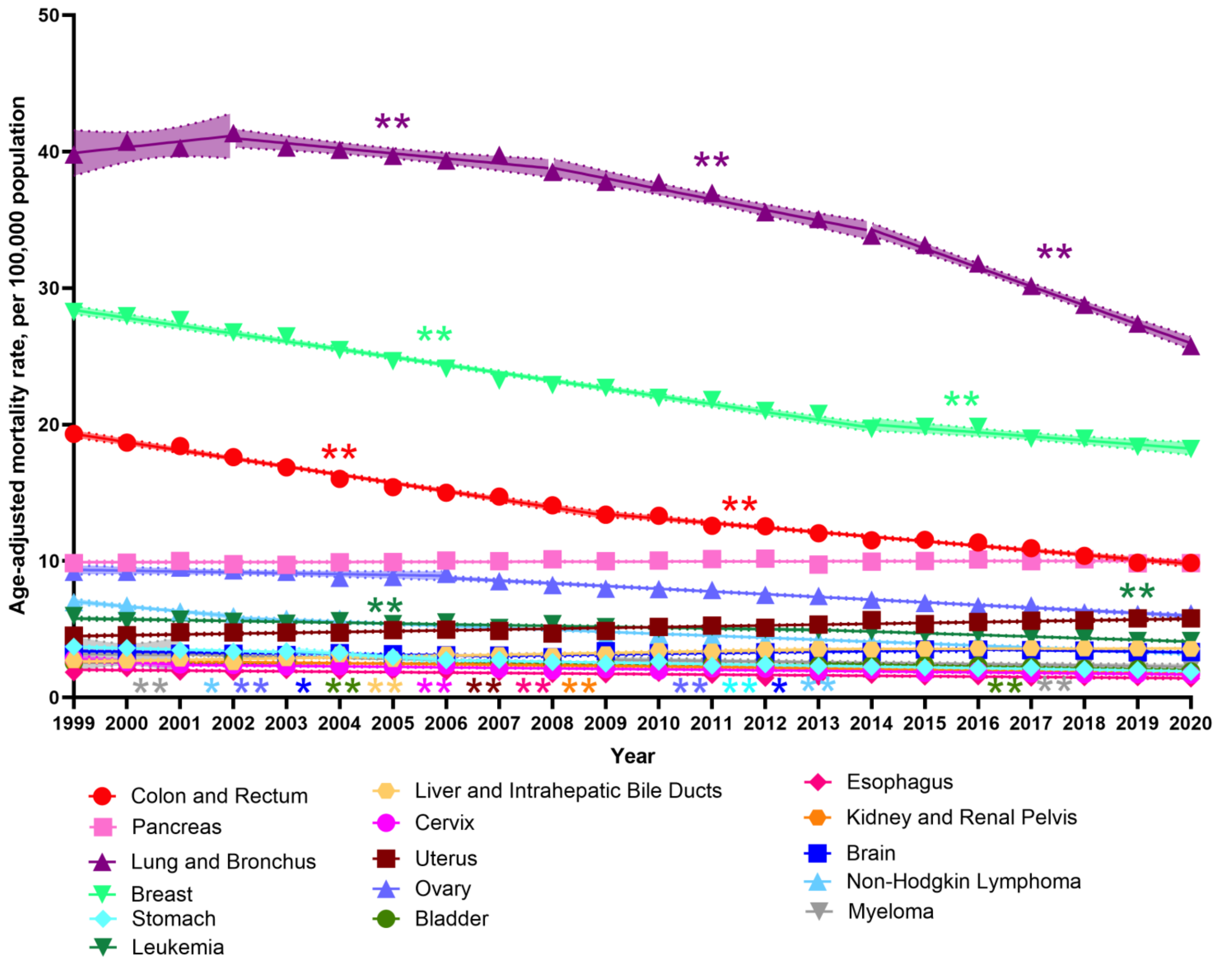

© 2024 Zhu DT et al. *JAMA Network Open*.

B. Midwest

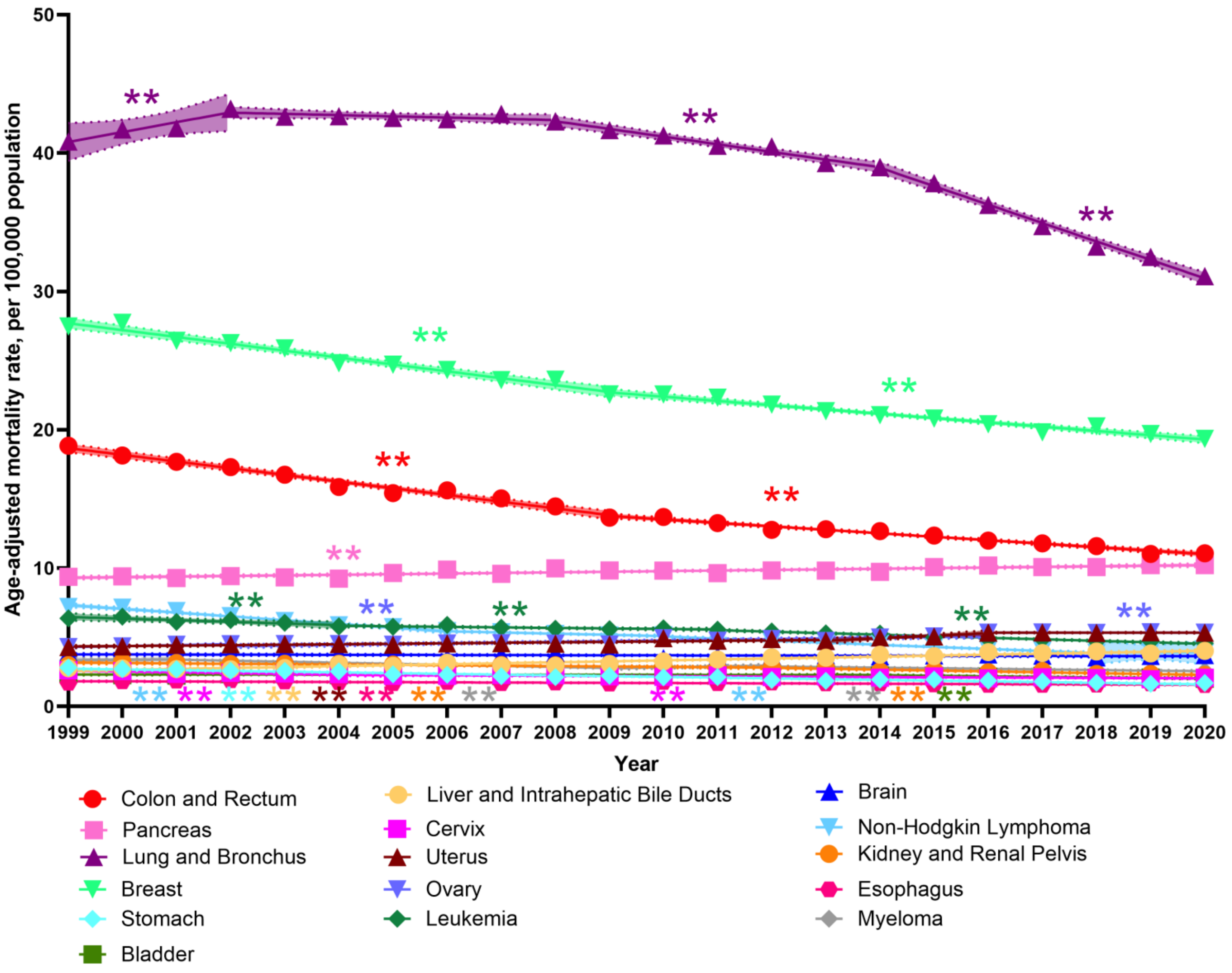

© 2024 Zhu DT et al. *JAMA Network Open*.

C. South

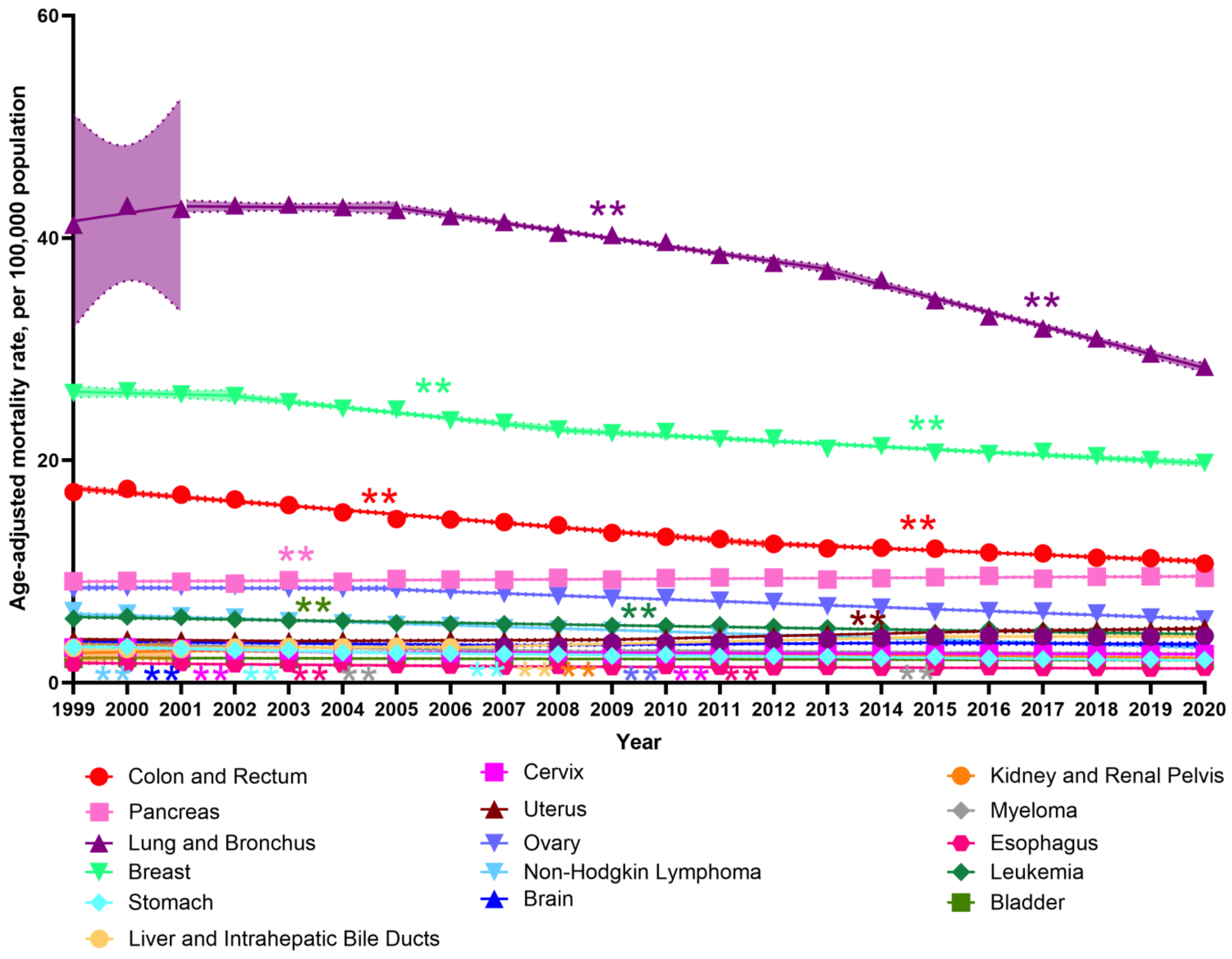

© 2024 Zhu DT et al. *JAMA Network Open*.

D. West

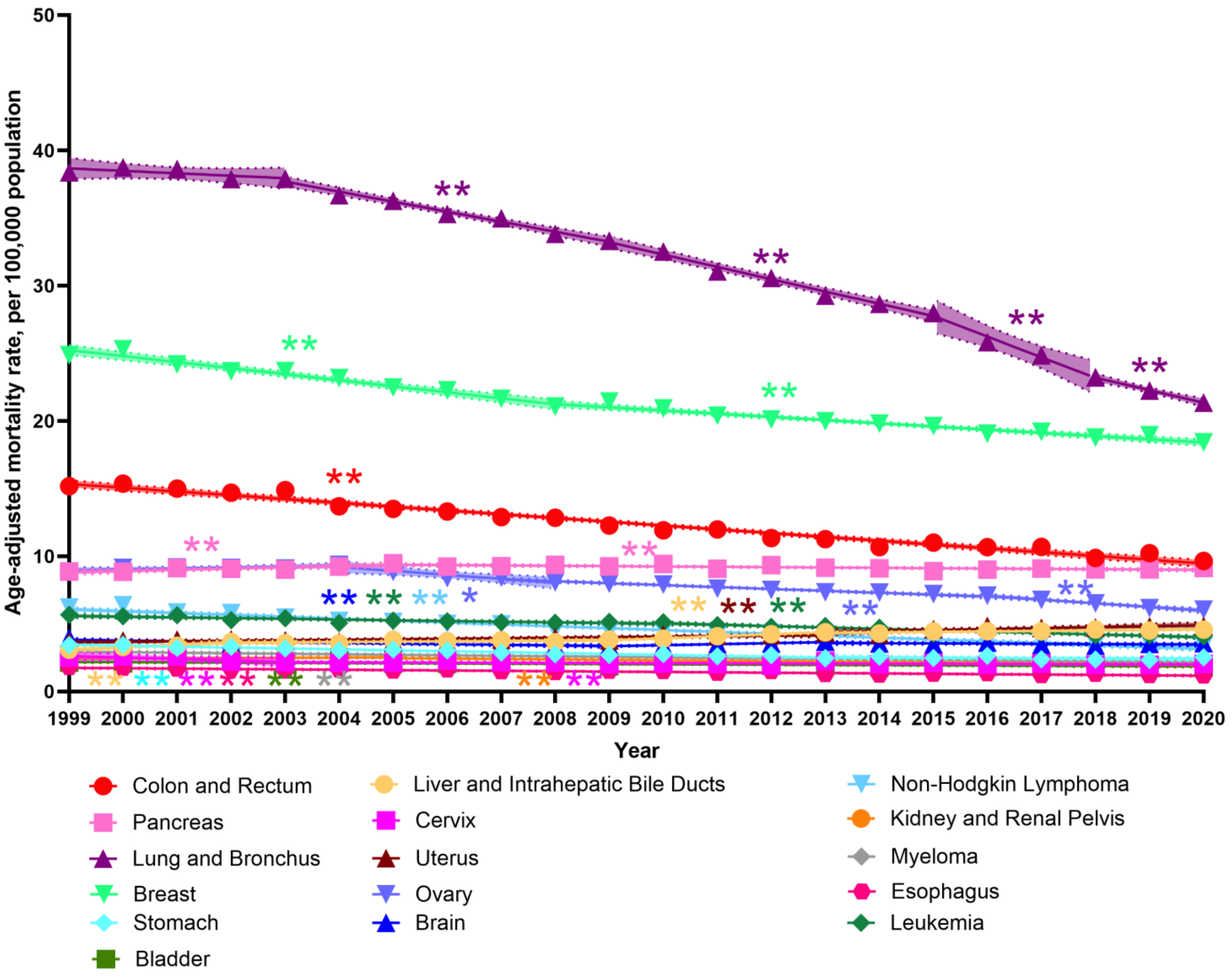

© 2024 Zhu DT et al. *JAMA Network Open*.

eFigure 3. Trends in Age-Adjusted Death Rates From 1999 to 2020 Among Asian American and Pacific Islander Women by Cancer Type and US Census Region. Observed rates are presented per 100,000 population and represented by markers. Modeled trends are represented by “---”. “ ** ” indicates the AAPC p-value is significant after Holm-Bonferroni correction. “ * ” indicates the p-value is <0.05, but not significant after Holm-Bonferroni correction. Confidence bands represent 95% confidence intervals.

© 2024 Zhu DT et al. *JAMA Network Open*.
