# Supplementary material for: US Cancer Mortality Trends Among Asian and Pacific Islander Populations
Source: JAMA Netw Open. 2024 Nov 4;7(11):e2442451. doi: 10.1001/jamanetworkopen.2024.42451 (PMC11581537; doi:10.1001/jamanetworkopen.2024.42451)
Supplement: Supplement 2. — Data Sharing Statement [file jamanetwopen-e2442451-s002.pdf]

Data Sharing Statement

Zhu. US Cancer Mortality Trends Among Asian and Pacific Islander Populations. *JAMA Netw Open*. Published November 04, 2024. doi:10.1001/jamanetworkopen.2024.42451

Data
Data available: Yes
Data types: Other (please specify)
Additional Information: All data used in this analysis are publicly available at https://wonder.cdc.gov/.
How to access data: All data used in this analysis are publicly available at https://wonder.cdc.gov/.
When available: With publication

Supporting Documents
Document types: None

Additional Information
Who can access the data: Immediately, at https://wonder.cdc.gov/.
Types of analyses: All data are publicly available at https://wonder.cdc.gov/.
Mechanisms of data availability: All data are publicly available at https://wonder.cdc.gov/.
